# The role of vaccination coverage, individual behaviors, and the public health response in the control of measles epidemics: an agent-based simulation for California

**DOI:** 10.1186/s12889-015-1766-6

**Published:** 2015-05-01

**Authors:** Fengchen Liu, Wayne T A Enanoria, Jennifer Zipprich, Seth Blumberg, Kathleen Harriman, Sarah F Ackley, William D Wheaton, Justine L Allpress, Travis C Porco

**Affiliations:** Francis I. Proctor Foundation, University of California, San Francisco, CA USA; Department of Epidemiology and Biostatistics, University of California, San Francisco, San Francisco, CA USA; California Department of Public Health, Immunization Branch, Richmond, CA USA; RTI Research Triangle Institute International, San Francisco, CA USA; Department of Ophthalmology, University of California, San Francisco, San Francisco, CA USA

## Abstract

**Background:**

Measles cases continue to occur among susceptible individuals despite the elimination of endemic measles transmission in the United States. Clustering of disease susceptibility can threaten herd immunity and impact the likelihood of disease outbreaks in a highly vaccinated population. Previous studies have examined the role of contact tracing to control infectious diseases among clustered populations, but have not explicitly modeled the public health response using an agent-based model.

**Methods:**

We developed an agent-based simulation model of measles transmission using the Framework for Reconstructing Epidemiological Dynamics (FRED) and the Synthetic Population Database maintained by RTI International. The simulation of measles transmission was based on interactions among individuals in different places: households, schools, daycares, workplaces, and neighborhoods. The model simulated different levels of immunity clustering, vaccination coverage, and contact investigations with delays caused by individuals’ behaviors and/or the delay in a health department’s response. We examined the effects of these characteristics on the probability of uncontrolled measles outbreaks and the outbreak size in 365 days after the introduction of one index case into a synthetic population.

**Results:**

We found that large measles outbreaks can be prevented with contact investigations and moderate contact rates by having (1) a very high vaccination coverage (≥ 95%) with a moderate to low level of immunity clustering (≤ 0.5) for individuals aged less than or equal to 18 years, or (2) a moderate vaccination coverage (85% or 90%) with no immunity clustering for individuals (≤18 years of age), a short intervention delay, and a high probability that a contact can be traced. Without contact investigations, measles outbreaks may be prevented by the highest vaccination coverage with no immunity clustering for individuals (≤18 years of age) with moderate contact rates; but for the highest contact rates, even the highest coverage with no immunity clustering for individuals (≤18 years of age) cannot completely prevent measles outbreaks.

**Conclusions:**

The simulation results demonstrated the importance of vaccination coverage, clustering of immunity, and contact investigations in preventing uncontrolled measles outbreaks.

**Electronic supplementary material:**

The online version of this article (doi:10.1186/s12889-015-1766-6) contains supplementary material, which is available to authorized users.

## Background

Measles is a highly infectious, acute viral illness that can cause severe pneumonia, diarrhea, encephalitis, as well as death in rare cases [1]. Measles cases continue to occur among susceptible individuals despite the elimination of endemic measles transmission in the United States as a result of importation [2,3]. The introduction of one imported measles case into a group or community with a large number of susceptible individuals can yield large outbreaks [4]. In California, the number of measles cases associated with visits to one of two Disney theme parks was 68 on January 23, 2015 (www.cnn.com/2015/01/23/health/california-measles-outbreak/index.html). In 2013, an intentionally unvaccinated adolescent returned to New York from London, United Kingdom while infectious with measles. As a result, the imported case led to the identification of 58 cases and six generations of measles infection among members of an orthodox Jewish community living in two neighborhoods in Brooklyn [5], resulting in the largest outbreak of measles in the United States since 1996 [5,6]. The outbreak was propagated by a few extended families that either refused the measles, mumps, and rubella (MMR) vaccine or delayed receipt of the vaccine. High population-level vaccination coverage outside of this community coupled with the insular nature of the community itself likely prevented further measles transmission [5]. Despite high vaccination coverage of a population, clustering of disease susceptibility due to personal beliefs and other exemptions to mandatory childhood vaccination can threaten herd immunity and impact the likelihood of disease outbreaks [7].

Previous researchers have used mathematical models to explore these empirical observations. The measles vaccination threshold to attain herd immunity in a population is believed to be approximately 90%--95%, but these estimates were based on models with relatively simple assumptions about contacts among individuals in the population, e.g., random mixing [8]. Mathematical models have examined infectious disease dynamics over specific contact networks [9] and household structure [10,11], particularly preferential mixing among small closely connected groups to show its effects on the reproductive numbers of infectious diseases [11]. In general, population-level disease dynamics are influenced by heterogeneous contact patterns [12]. Furthermore, clustering may decrease the size of epidemics, but also may decrease the epidemic threshold, making it easier for a disease to spread [13]. Furthermore, outbreaks can occur even with very high vaccination rates as the clustering of vaccination increases [8].

With each identified measles case, public health departments investigate the close contacts of the case in order to identify anyone who may have been exposed while the case was infectious. If susceptible close contacts are identified, contact tracing efforts can lead to public health interventions (i.e., giving exposed contacts post-exposure vaccine or post-exposure immune globulin) with the hope of minimizing the chance of disease development. In addition, exposed individuals are asked to monitor their symptoms for at least one incubation period, and report any symptoms to public health authorities, should they develop symptoms consistent with measles. However, people do not always comply with public health interventions or recommendations. Also, public health authorities cannot always find identified contacts, or do not always reach susceptible contacts soon enough in order for post-exposure prophylaxis to be effective; contacts would be placed in quarantine in these instances. With vaccination coverage in the community, clustering of susceptibility, individual behaviors, and contacts occurring over networks of individuals, one question that remains is under which circumstances do contact tracing activities and the associated interventions given by a local or state health department help to control measles epidemics? The purpose of our study was to identify the characteristics of population vaccination coverage, clustering of susceptibility, individuals’ behaviors, and the public health response that impact the occurrence of measles epidemics.

## Methods

### Ethics statement

The project received a determination of not being research involving human subjects when reviewed by the Committee for the Protection of Human Subjects at the California Department of Public Health.

### Model structure

We developed an agent-based simulation model of measles transmission after the introduction of one index case into a population in order to study the effects of contact tracing and public health interventions on the occurrence of measles epidemics. The agent-based simulation model has two main components: (1) the agent-based simulation model of measles transmission using the Framework for Reconstructing Epidemiological Dynamics (FRED) [14,15], and (2) a synthetic population of individuals adapted from the Synthetic Population Database maintained by RTI International [16]. In this section, we describe the various features of our model: the natural history of measles, the synthetic population, the contact network and transmission assumptions, clusters of immunity, contact investigations, public health interventions, and the analysis of the results. Briefly, all individuals in the synthetic population were in a social network and were given characteristics consistent with the U.S. Census and the California Department of Finance population estimates (www.dof.ca.gov), and measles could be transmitted through interactions among individuals in this network. The model simulated different levels of vaccination coverage, different levels of immunity clustering, and delays in contact investigations. We examined the effect of changes in the various features of the model on changes in the number of secondary cases, the total outbreak size (i.e., the total number of diagnosed cases), and the probability of uncontrolled outbreak scenarios over one year (i.e., chains of transmission continuing longer than one year).

### Natural history of measles

The natural history of measles was modeled as follows: an infected individual was assumed to be infectious for approximately nine days (four days before rash onset through four days after the onset of rash [1,17]) after a latent period of approximately eight days [18]. A ten-day symptomatic period [19-24] began 8 to 14 days after exposure [25,26] with rash onset beginning 7 to 19 after exposure [27]. An individual who recovered from an infection was no longer susceptible to subsequent infection. In the agent-based model, we used a standard SEIR model (susceptible-exposed/latent-infectious-recovered) [28] to represent an individual’s health states.

### Synthetic population

Our model incorporated a synthetic population generated from a Synthetic Population Database developed by RTI International [16] that represented every individual in a specific geographic region. The synthetic population was generated based on the U.S. Census and the California Department of Finance population estimates, and each individual had associated demographic information (e.g., age, sex) and locations for social activity (e.g., household, neighborhood, and possibly school or workplace). To reflect measles transmission among preschool-aged children [29], we added daycares to the synthetic population (using daycare data from the California Department of Social Services’ Community Care Licensing Division: www.ccld.ca.gov) according to location information for daycare-aged children. Further daycare details in the synthetic population are given in the Additional file 1.

### Contact network and transmission

The simulation of measles transmission was based on interactions among individuals in different places: households, schools, daycares, workplaces, and neighborhoods. Depending on the assigned locations of social activity based on the synthetic population data, each individual visited his or her household and neighborhood on each simulated day. The individual could have visited his/her school, daycare, or workplace if the individual was a student, a daycare-aged child or employed, respectively. In addition, the individual could interact with other individuals who shared the same activity locations for the same simulated day.

For an infectious individual, the number of contacts on a given day depends on the contact rate per day (the time unit of simulation is one day) in each of the locations the infectious individual has visited, as well as the probability of transmission per contact. The contact rate per day depends on the type of place of the contact, as shown in Table 1. We assume that, on a given day, a child attending a daycare may have 3 to 20 random contacts with other children who are in the same daycare; similarly, a student attending a school may randomly contact 3 to 20 schoolmates (base on the child-staff ratio for daycares in California: www.daycare.com/california, and the average class size in California public schools: www.cde.ca.gov/ds/sd/dr/cefteachavgclssize.asp); a worker may contact 0 to 7 co-workers (according to the RTI’s synthetic population used by the model, 61.4% workplaces have the number of workers ≤7); each individual may have 0.5 to 7 neighborhood contacts; each individual contacts each of his/her household members with a probability ranging from 0.01 to 1. We assume that the transmission probability per contact is very high, as measles is a highly contagious disease [30].

**Table 1:** **Parameters**

**Parameter**	**Initial value**	**Lower**	**Upper**	**Interpretation**	**Reference**
*V*: vaccination coverage	0.95	0.85	1	vaccination coverage rate for individuals aged less than or equal to 18 years	[32-34]
*Ω*: immunity clustering	0.5	0	1	level of immunity clustering for individuals aged less than or equal to 18 years in a household	
Household contact probability	0.46	0.01	1	contact probability per day between any two household members	
Neighborhood contact rate	3 person/day	0.5	7	contact rate per day in neighbourhood	assume
Workplace contact rate	3 person/day	0	7	contact rate per day in workplace	synthetic population
School contact rate	9 person/day	3	20	contact rate per day in school	www.cde.ca.gov/ds/sd/dr/cefteachavgclssize.asp
Daycare contact rate	9 person/day	3	20	contact rate per day in daycare	www.daycare.com/california
Household transmission probability	0.99	0.99	0.99	transmission probability per contact in household	[30]
Neighborhood transmission probability	0.99	0.99	0.99	transmission probability per contact in neighborhood	[30]
Workplace transmission probability	0.99	0.99	0.99	transmission probability per contact in workplace	[30]
School transmission probability	0.99	0.99	0.99	transmission probability per contact in school	[30]
Daycare transmission probability	0.99	0.99	0.99	transmission probability per contact in daycare	[30]
Vaccine efficacy	0.99	0.99	0.99	efficacy of two doses of vaccine for measles, mumps and rubella	[35,36]
Trace probability	1	1	1	probability that an individual is traceable	assume
Intervention delay	1 day	1	3	intervention delay for contacts of an index case	(J. Zipprich, pers. commun.)
Contact tracing delay	1 day	1	3	delay for tracing a contact from an infectious case	assume
Self report delay	2 day	1	6	delay between the first day of symptom of a case and the day the case visits hospital	assume
Cooperation probability	1	1	1	probability that an individual is cooperative to accept public health interventions	assume
Contact finding probability	1	0.7	1	probability that a contact of an infectious case can be traced	assume
Post-exposure prophylactic vaccine efficacy	0.93	0.92	0.95	efficacy of post-exposure prophylactic vaccine for measles, mumps and rubella	[35,36]
Post-exposure prophylactic immune globulin efficacy	0.75	0.6	0.9	efficacy of post-exposure prophylactic immune globulin	[60-62]
Home quarantine probability	0.97	0.9	1	probability that an individual who is recommended to stay home until recovery will follow the recommendation	assume
Home stay probability	0.61	0	1	probability that a case decides (on each day of the symptomatic period of the case) to stay home until recovery	assume

Whenever an infective individual contacts another individual, the chance that disease transmission will actually occur depends on the immunity level of the contact. The immune status of each simulated individual is a function, in part, of the age-specific vaccination coverage and the efficacy of vaccine (see Section: Clusters of immunity). Once the susceptible individual is infected, the individual is infectious for approximately nine days [1,17] starting the day after the latent period, which is approximately eight days long [18]. The probability distributions of the numbers of days spent in the latent period and infectious period are given in the Additional file 1. For each transmission episode, data about the episode (i.e., date, infector, infectee, and location) are recorded by the simulation program, and this transmission record is used in the simulations involving contact tracing and public health interventions (see Section: Contact investigation and public health intervention).

Measles transmission is initiated by introducing one randomly selected index case, whose age is consistent with the observed age distribution of measles cases in California, into a highly vaccinated population; the initialization of vaccination will be discussed in Section Clusters of immunity. After the latent period, the index case may transmit measles by contacting susceptible individuals in different locations (household, neighborhood, school, daycare, or workplace settings) on each day of the infectious period. Each new infectious individual caused by the index case may then transmit measles via the individual’s contact network during his or her infectious period. To model an infected individual’s daily behavior during the symptomatic period, we assume that the individual may decide to stay home with a specified probability (ranging from 0 to 1, Table 1) on each day of the symptomatic period until recovery.

To reflect the difference of social activities between weekdays and weekends during the simulation, schools and daycares are assumed to be closed on weekends. Approximately 34% of workers are randomly chosen to continue visiting their workplaces on weekends (based on data from the Bureau of Labor Statistics: www.bls.gov/news.release/atus.nr0.htm) and the number of neighborhood contacts per day on weekends is assumed to increase by 50% [15].

### Clusters of immunity

Despite high vaccination coverage, clustering of disease susceptibility due to personal beliefs and other exemptions to mandatory childhood vaccination can threaten herd immunity and impact the likelihood of disease outbreaks [7]. Salathe *et al.* [31] showed that the effect of clustered susceptible individuals on disease outbreak probabilities is strong when the vaccination coverage is close to the level required to provide herd immunity under the assumption of random mixing.

To model the clusters of susceptible children in a highly vaccinated population (i.e., vaccination coverage of two-dose of MMR was assumed to range between 95% to 99% (V) for children less than or equal to 18 years of age [32-34] and 92.4% to 97.9% for individuals greater than 18 years of age [34]), we proceed as follows. On one extreme, we simply assume random vaccination of children; under this assumption, the vaccination status of each child is independent of the status of all other children in the household; we denote the coverage level by V. At the other extreme, we assume that while the overall coverage is still V, all children in a given household have the same vaccine status. In the model, we interpolate between these two extremes, with Ω = 0 representing random vaccination, and Ω = 1 denoting completely positive assortative vaccine status. The interpolation formula is given in the Additional file 1; values of Ω between 0 and 1 represent intermediate configurations between random vaccination and completely positive assortative vaccination of children. For each synthetic household, we assign vaccination status to each household member in this way to initialize the clusters of immunity in the synthetic population with a given level of clustering, Ω, (assuming that the efficacy of two doses of MMR vaccine is 99% [35,36], Table 1). The simulation of measles transmission discussed in Section Contact network and transmission is in a highly vaccinated and susceptibility-cluster-specific population. Note that detailed age-specific vaccine coverage data by household are not available.

### Contact investigation and public health intervention

For an identified case, the public health department cannot know all the contacts of the case, and may not be able to find every contact of the case. To simulate a contact investigation, which may depend on the type of place and the probability that the contacts of an infectious case can be traced, we proceed as follows. First, we assume that neighborhood contacts are not traceable. However, each of the contacts from the household, school, daycare, and workplace settings can be identified by the infectious individual with some specified probability. In addition, the identified contact can be found by the public health department with a probability ranging from 0.7 to 1 (in Table 1).

For an individual who is traced from an infectious case by a contact investigation and receives a public health intervention, several days may have elapsed since exposure. For an infectious individual (either an index case or a case not prevented by the contact tracing and public health interventions) who visits the doctor and is confirmed as a measles case, there may have been several days since the first day of symptoms. To simulate a contact investigation, we assume there are three delays: (i) the self report delay for an infectious individual, i.e., the time between symptoms and diagnosis, (ii) the intervention delay for contacts of an index case, and (iii) the delay for finding a contact of an infectious case.

The *self report delay* for an infectious individual, i.e., the time between symptoms and diagnosis, is assumed to be between one and six days (J. Zipprich, personal communication, Table 1). The *intervention delay* for contacts of an index case occurs if the infectious individual is an index case, there may be an additional one to three days (*intervention delay* in Table 1), which is the time between diagnosis and initiation of contact investigation. If the infectious individual is not the index case but instead is a subsequent case, we assume a further round of contact investigation is inaugurated without delay. The delay for finding a contact of an infectious case occurs when the health department may need an additional one to three days (*contact tracing delay* in Table 1) to obtain the names of the infectious individual’s contacts, trace them, test immunity and provide interventions.

For an individual who is traced by a contact investigation and receives a public health intervention, to simulate the delay from the first day of symptoms to the day of receiving a public health intervention, we assume that there are four scenarios:

- If the individual is an index case, the individual receives the public health intervention at a time ranging from 2 to 9 days after the first day of symptoms, i.e., the sum of the self report delay and the intervention delay;
- If the individual is the infector and an infectious case but not an index case, the individual receives the public health intervention at a time ranging from 1 to 6 days (the self report delay) after the first day of symptoms;
- If the individual is the infectee and the infector is an index case, the individual receives the public health intervention at a time ranging from 3 to 12 days (the sum of three delays) after exposure;
- If the individual is the infectee and the infector is an infectious but not an index case, the individual receives the public health intervention at a time ranging from 2 to 9 days (the sum of the self report delay and the contact tracing delay) after exposure.

According to California Department of Public Health [37], post-exposure prophylactic (PEP) MMR vaccine may be given within 72 hours of exposure to persons ≥ 6 months of age. Post-exposure prophylactic immune globulin (IG) may be given within 6 days to exposed persons of any age, though persons weighing less than 30 kg are prioritized. Home quarantine may be recommended for exposed persons contacted seven days or more from exposure. To simulate these three public health interventions (PEP MMR, PEP IG and home quarantine), we assume that one dose of PEP MMR is given to an individual if it has not been more than three days since the first day of exposure, the individual is willing to accept PEP MMR, and the individual’s age is greater than one year of age (using one year as the cutoff is consistent with the public health department’s practice). We assume that PEP IG is given to an individual if the individual’s age is ≤ 1 year and it has not been more than six days since the first day of exposure, or if the individual’s age is >1 year, the individual is cooperative, and it has been more than three days but not more than six days since exposure. For an individual who is not willing to accept PEP MMR or PEP IG, or if it has been more than six days since the first day of exposure, we assume that home quarantine is recommended.

Briefly, we simulated contact tracing activities and public health interventions in four steps:

- Record all contacts. On each simulated day, the simulation of measles transmission (Section Contact network and transmission) records the information for every contact, including the day when a transmissible contact occurs, the infector (the identity of the infectious individual), the infectee (the identity of the individual contacted by the infector), and the place (location/setting where transmissible contact occurred).
- Trace contacts. When a case is diagnosed, the case is interviewed and contacts elicited, which is modeled by choosing a random sample of the true contacts (excluding neighborhood contacts).
- Model public health interventions for elicited contacts (including simulated time delays and loss to follow-up). For each elicited contact, a random Bernoulli trial is simulated with the contact finding probability, and if the value is 1 (“success”), then a random waiting time is added. The individual will then be contacted for any interventions at that time in the future (a random variable with an exponential distribution). Only contacts within the last seven days are elicited (by assumption).For an individual who has been given any intervention in a previous contact investigation, we do not schedule another public health intervention within seven days since the last intervention.
- Give public health interventions. First, not all individuals cooperate with investigators; we, therefore, generate a random Bernoulli trial with the cooperation probability. If the result is 1 (“success”), then we determine how much time has elapsed since transmission for each individual with a scheduled public health intervention. Either PEP MMR, PEP IG, or a home quarantine recommendation is provided, depending on the elapsed time.

### Analysis

We analyzed the model in two steps. First, we selected a Latin Hypercube sample [38,39] of parameters chosen uniformly from the parameter ranges given in Table 1, and simulated one year of transmission with contact investigation and public health intervention. For each parameter set, we introduced a single index case into the synthetic population, and determined the total number of new cases diagnosed during the year, and whether or not active transmission was still occurring at the end of the year. In many cases, transmission chains die out rapidly, while for other simulated scenarios, transmission continues to occur. For each parameter set, we replicated the epidemic 256 times (2^8^, chosen arbitrarily, but sufficient to ensure a 50% proportion has an estimated standard deviation less than 5%). Thus, for each scenario (parameter set), we determined (1) the number of secondary cases, (2) the outbreak size, and (3) the probability that there were over 500 cases within the year (uncontrolled transmission or “escape” probability). For each replication, we also calculated the number of symptomatic cases directly caused by the index case (allowing the expected number of secondary cases per introduction to be estimated). For a given parameter set, the simulation also monitored the outbreak size for each of 256 iterations; if the outbreak size was more than 500, the iteration was classed as uncontrolled transmission. The probability of uncontrolled transmission for each scenario was estimated as the number of introductions leading to uncontrolled transmission, divided by the total number of introductions (256 in each case). Second, we plotted the results after one year of simulated measles transmission with contact investigations and public health interventions for selected parameter sets.

## Results

### Sensitivity analysis

To determine which parameters were the most important in determining the number of secondary cases, the outbreak size, and the probability of uncontrolled outbreak in a one year of measles transmission, we selected a Latin Hypercube sample of 1024 different parameter sets from the parameter ranges indicated in Table 1. From each, the number of secondary cases, the number of outbreak size, and the probability of uncontrolled measles outbreaks (over one year) were computed from 256 replications. For definiteness, we chose Humboldt County in California (population size 118,261). The simulation was started by introducing one index case who was randomly selected according to the observed age distribution of measles cases in California. We then computed the partial rank correlation coefficient (PRCC) [40,41] between each input parameter and each of three simulation outcomes (the number of secondary cases, the total outbreak size and the probability of uncontrolled measles outbreaks in 365 days). When the PRCC is close to zero, the value of the parameter has little relation to the simulation output; when the PRCC is close to +1 or −1, the value of the parameter is highly important in determining the simulation output.

### Scenario analysis

We chose 4 parameters (PRCC ≥ 0.1, in Table 2), which are (1) vaccination coverage, (2) level of immunity clustering (Ω), (3) intervention delay, and (4) contact finding probability to explore factors which contribute to the successful control of measles (i.e., the probability of uncontrolled measles outbreak). In this section, we examine (1) the combinations of vaccination coverage and the level of immunity clustering, (2) the combinations of intervention delay and contact finding probability based on different levels of immunity clustering and vaccination coverage, (3) the combinations of vaccination coverage and contact rates in the neighborhood, and (4) the combinations of vaccination coverage and contact rates in all places. We report the probability of escape, or the mean outbreak size (when no scenarios showed a total number of cases exceeding 500 by one year after measles introduction). Due to the critical threshold level for the fraction of susceptible individuals below which introduction of infections can only lead to minor outbreaks and above which minor and major outbreaks can occur [42], we present the probability of uncontrolled outbreaks for scenarios in which minor and major outbreaks occurred and total outbreak size for scenarios that only led to minor outbreaks [43].

**Table 2:** **PRCC of parameters with secondary cases, outbreak size and probability of uncontrolled measles outbreak in 365 days**

**Name**	**Secondary Case**	**Outbreak size**	**Outbreak probability**
V: vaccination coverage	−0.4464	−0.4896	−0.3142
*Ω*. : immunity clustering	0.3364	0.4967	0.3612
Household contact probability	−0.09997	−0.05333	−0.05513
Neighborhood contact rate	0.8834	0.887	0.7236
Workplace contact rate	0.07722	0.0876	0.08536
School contact rate	0.1087	0.1064	0.05295
Intervention delay	0.1884	0.1937	0.1561
Contact tracing delay	0.06729	0.04466	0.0269
Self report delay	0.4406	0.4352	0.3392
Contact finding probability	−0.135	−0.1961	−0.09143
Post-exposure prophylactic vaccine efficacy	−0.005373	−0.02823	−0.04407
Post-exposure prophylactic immune globulin efficacy	−0.09336	−0.08637	−0.05275
Home quarantine probability	−0.0598	−0.06094	−0.05357
Home stay probability	−0.9049	−0.9119	−0.8553
Daycare contact rate	0.01553	0.003767	0.0249

### Effects of vaccination coverage and the level of immunity clustering

**Figure 1:**
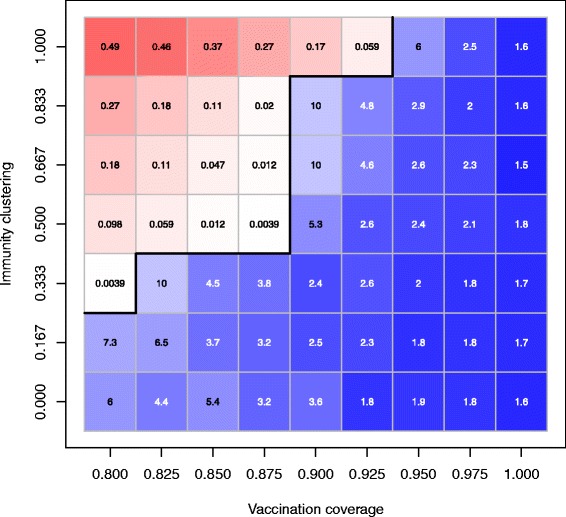
Effects of vaccination coverage and clustering of immunity on the control of measles epidemics. For each combination of vaccination coverage V and the level of immunity clustering Ω (all other parameters’ values are shown in Table 1), we ran 256 iterations to obtain the outbreak size and the uncontrolled outbreak probability in 365 simulated days. The combinations with which the simulated uncontrolled outbreak probabilities >0 are represented by red cells and scaled from light red (lower uncontrolled outbreak probability) to dark red (higher uncontrolled outbreak probability); the values of simulated uncontrolled outbreak probabilities are shown in red cells. The combinations without uncontrolled outbreaks (the simulated uncontrolled outbreak probabilities =0) are shown by blue cells and scaled from light blue (higher outbreak size) to dark blue (lower outbreak size); the values of simulated outbreak sizes are shown in blue cells. The frontiers between adjacent combinations with and without uncontrolled outbreaks are shown by the black lines. These simulations suggest that the vaccination coverage is important in the control of measles epidemics (the higher vaccination coverage, the lower the probability of uncontrolled measles outbreaks and the smaller outbreak size); for a given vaccination coverage, a lower level of immunity clustering (i.e., the lower the chance of unvaccinated individuals clustered together in a household) may have better control of measles epidemics.

For combinations of vaccination coverage ranging from 0.8 to 1 and the level of immunity clustering ranging from 0 to 1 (all other parameter values are shown in Table 1), the results are shown in Figure 1. When at least one escape scenario occurred (out of 256 trials), the result is shown in red; when there were no escape scenarios, the mean outbreak size is shown in blue; the border between an escape scenario and a no escape scenario is shown in black. Figure 1 shows that the vaccination coverage required to prevent measles outbreaks was approximately 95% for all levels of immunity clustering. For the scenario with the lowest level of immunity clustering, the threshold vaccination coverage was 80%. For the combinations with which there were no measles outbreaks, Figure 1 also shows that the outbreak size dramatically decreased with an increase in vaccination coverage for a given level of immunity clustering. These simulations simply illustrate the importance of high vaccination coverage and that clustering of unvaccinated individuals is unfavorable for disease control. The results assumed the contact rates, self-report delays, intervention delays for contacts of the index case, and the probability that a contact can be traced were constant (Table 1).

### Effects of intervention delay and contact finding probability based on different levels of immunity clustering and vaccination coverage

**Figure 2:**
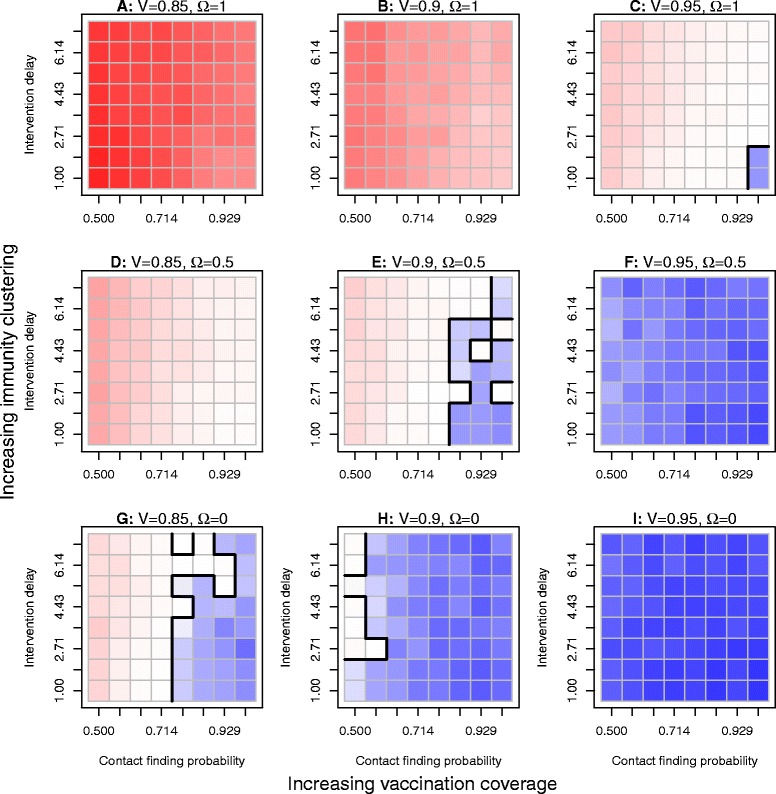
Effects of intervention delays and the contact finding probability on the control of measles epidemics. We simulated the main outcomes for each combination of the intervention delay for contacts of the index case and the contact finding probability under 9 combinations **(A**, **B**, **C**, **D**, **E**, **F**, **G**, **H and I)** of vaccination coverage (V= 0.85, 0.9 and 0.95) and the level of immunity clustering (Ω=0, 0.5 and 1). Cells in red indicate the combinations with which the simulations had uncontrolled outbreaks with the uncontrolled outbreak probabilities ranging from low to high. Cells in light blue show the combinations without uncontrolled outbreaks but with higher outbreak sizes than the combinations without uncontrolled outbreaks represented by dark blue cells. The frontiers between adjacent combinations with and without uncontrolled outbreaks are shown by the black lines. The results in **(A)**, **(B)** and **(C)** suggest that increasing vaccination coverage levels and the contact finding probability while reducing intervention delays may reduce the uncontrolled outbreak probability. However, measles outbreaks may not be prevented with the highest level of immunity clustering. The results in **(D)**, **(E)** and **(F)** suggest that scenarios with a lower level of immunity clustering, increasing vaccination coverage and contact finding probability, and reducing intervention delays have smaller uncontrolled outbreak probabilities than the highest level of immunity clustering (shown in Figures 2 **(A)**, **(B)** and **(C)**), and 95% of vaccination may be enough to prevent measles outbreak which is consistent with the result shown in Figure 1 (the cell with V=95% and Ω=0.5); when V=90%, measles outbreaks may be prevented by the combinations of low intervention delays and a high contact finding probability. The results in **(G)**, **(H)** and **(I)** suggest that with the lowest level of immunity clustering, increasing vaccination coverage may dramatically reduce measles outbreaks.

To expand further on the relationship between vaccination coverage and immunity clustering and their role in the control of measles outbreaks, we examined the influence of two parameters related to contact investigations, intervention delay for contacts of an index case and the contact finding probability, on the occurrence of measles outbreaks. In Figure 2, we present the results of our findings for three levels of vaccine coverage (V = 0.85, 0.90, and 0.95 in the three columns) and three levels of immunity clustering (Ω=0, 0.5, and 1.0 in the three rows). For each coverage-clustering combination, we show the results of our simulations across values of the contact finding probability (x-axis ranging from 0.50 to 1.0) and the intervention delay for contacts of the index case (y-axis ranging from 1 to 7 days). In the setting with the lowest vaccination coverage and the highest level of immunity clustering (V=0.85 and Ω=1.0, Figure 2(A)), we found that escape scenarios could occur for all values of the intervention delay and the contact finding probability, and thus report the probability of escape scenarios denoted in red. In the setting with the highest vaccination coverage and the lowest level of immunity clustering (V=0.95 and Ω=0.0, Figure 2(I)), we obtained no uncontrolled outbreaks, and report the total outbreak size for all values of the intervention delay and the contact finding probability (shown in blue). The settings where the levels of the intervention delay and the contact finding probability become important were in Figure 2(C), (E), and (G), when the coverage-clustering combinations were either both high (Figure 2(C)), both moderate (Figure 2(E)), or both low (Figure 2(G)). For these three settings, the level of intervention delay for contacts of the index case and the probability a contact can be found determines whether we have controlled or uncontrolled measles outbreaks. Even with stochastic simulation error, higher contact finding probabilities with lower intervention delays lead to controlled measles outbreaks.

However, Figure 2 does not show the effects of the speed of contact investigation on the uncontrolled probability and the outbreak size. To study the effects of the speed of contact investigations, we varied the self report delay, the intervention delay and the contact tracing delay and found that the uncontrolled outbreak probabilities and the outbreak sizes were reduced by increasing the speed of contact investigation (as shown in Table 3). In general, the speed of contact investigations does affect how well it contributes to disease control.

**Table 3:** **Effects of the speed of contact investigation on outbreak sizes and uncontrolled outbreak probabilities**

**Uncontrolled outbreak probability**	**Outbreak size**	**Self report delay**	**Intervention delay**	**Contact tracing delay**	**Vaccination coverage**	**Clustering**
0.215	749.4	6	7	3	0.95	1
0.191	636	6	3	3	0.95	1
0.098	152	4	2	2	0.95	1
0	2.8	1	1	1	0.95	1
0.152	371.6	6	7	3	0.9	0.5
0.148	344.8	6	3	3	0.9	0.5
0.054	69.8	4	2	2	0.9	0.5
0	3.3	1	1	1	0.9	0.5

### Effects of vaccination coverage and contact rate in neighborhood

**Figure 3:**
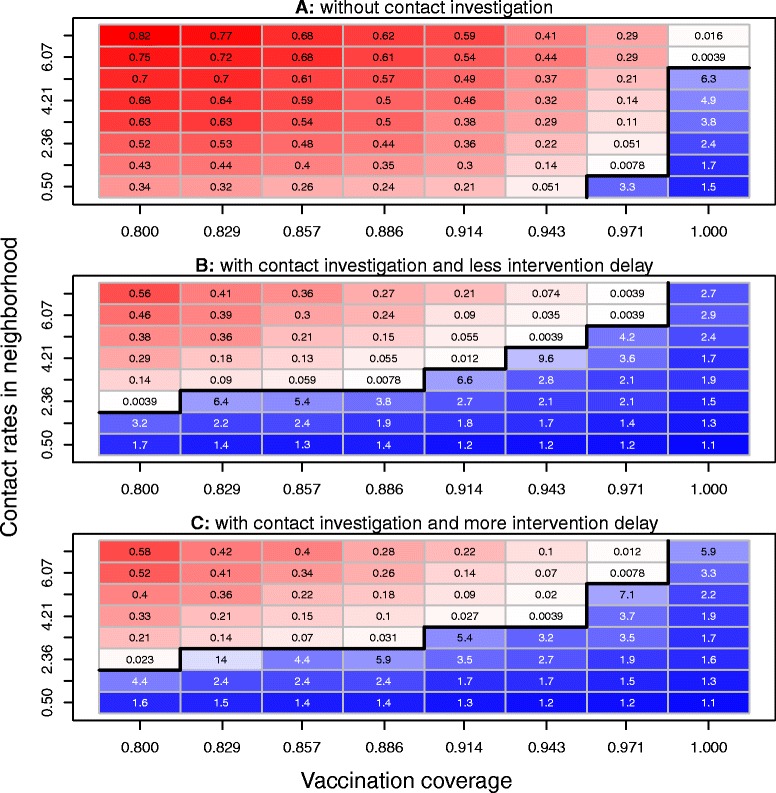
Effects of vaccination coverage and the contact rate in neighborhoods on the control of measles epidemics. We used the parameters’ values in Table 1 and combinations of vaccination coverage and contact rates in the neighborhood, and ran 256 iterations for each combination to simulate the uncontrolled outbreak probability and the outbreak size in 365 days under three scenarios: (**A**) without contact investigations, (**B**) with contact investigations and less intervention delay for contacts of the index case, and (**C**) with contact investigations and more intervention delay for contacts of the index case. For each of the scenarios **A**, **B**, and **C**, red cells show the combinations with uncontrolled outbreaks (the simulated uncontrolled outbreak probabilities >0; light red cells indicate combinations with lower uncontrolled outbreak probabilities; dark red cells indicate combinations with higher uncontrolled outbreak probabilities); and blue cells represent the simulated outbreak sizes of the combinations without uncontrolled outbreaks (the simulated uncontrolled outbreak probabilities =0), and scale the outbreak sizes from low (dark blue) to high (light blue). The frontiers between adjacent combinations with and without uncontrolled outbreaks are shown by the black lines. These simulations suggest: contact investigation plays an important role in preventing measles uncontrolled outbreaks and reducing the total outbreak size; with contact investigations, reducing the contact rates in the neighborhood may lower the vaccination coverage required to prevent uncontrolled measles outbreaks; with contact investigations and the highest vaccination coverage, measles outbreaks may be prevented even with very high contact rates in the neighborhood; less intervention delays for contacts of the index case may help contact investigations reduce the probability of uncontrolled measles outbreaks and the total outbreak size.

The PRCC value for the neighborhood contact rate (in Table 2) indicates that the transmission in the neighborhood setting is highly important in determining the probability of uncontrolled measles outbreaks and the total outbreak size because of the assumption that contact tracing does not happen in the neighborhood (i.e., people are not likely to identify the people they encounter in the neighborhood setting). We used the parameters’ values in Table 1 and combinations of vaccination coverage (ranging from 0 to 1) and the neighborhood contact rate (ranging from 1 to 7) to study their effects on the uncontrolled measles outbreak probability and total outbreak size. The simulation for each combination was run under three scenarios: (1) without contact investigation, (2) with contact investigation and little intervention delay for contacts of the index case, and (3) with contact investigation and more intervention delay for contacts of the index case. For the scenario without contact investigation, Figure 3(A) shows that more neighborhood contacts caused more outbreaks; increasing vaccination coverage could reduce the number of measles cases, but even with the highest vaccination coverage (100%), it still could not completely prevent the measles outbreak for high contact rates in neighborhood. For the scenario with contact investigation and less intervention delay for contacts of the index case, as shown Figure 3(B), with lower contact rates in the neighborhood, measles could be controlled even with 80% vaccination coverage; and 100% of vaccination coverage could prevent measles outbreaks for all contact rates in the neighborhood. For the scenario with contact investigations but with more intervention delays for contacts of the index case, Figure 3(C) shows that measles outbreaks were prevented by the combinations of low contact rates in the neighborhood with all vaccination coverage levels, and the combinations of the highest vaccination coverage level with all contact rates in neighborhood. Most of the uncontrolled outbreak probabilities and outbreak sizes were higher than those of the scenario with contact investigation and less intervention delays for contacts of the index case, i.e., there is less of a chance for a traced contact to receive PEP MMR or PEP IG with more intervention delays. By comparing the same combinations of the scenario without contact investigation in Figure 3(A), the probabilities of uncontrolled outbreaks and the outbreak sizes among the other two scenarios in Figures 3(B) and (C) were dramatically reduced by contact investigations. These simulations suggest: (1) contact investigation plays an important role in preventing measles outbreaks and reducing the outbreak size; (2) with contact investigations, reducing the contact rate in the neighborhood may lower the vaccination coverage required to prevent measles outbreaks; (3) with contact investigation and the highest vaccination coverage, measles outbreaks may be prevented even with very high contact rates in neighborhood; and (4) less intervention delays for contacts of the index case may help contact investigations reduce the probability of uncontrolled measles outbreaks and the outbreak size.

### Effects of vaccination coverage and contact rates in all places

**Figure 4:**
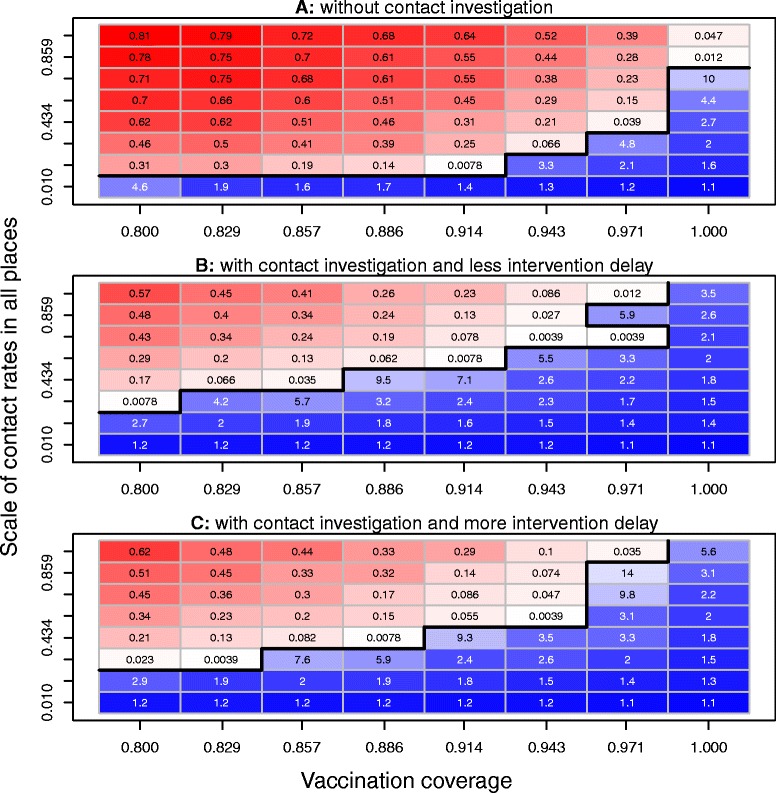
Effects of vaccination coverage and contact rates in all places on the control of measles epidemics. We used a scale parameter ranging from 0 to 1 to scale contact rates in school, daycare, workplace, household and neighborhood. For each combination of vaccination coverage V and the scale of all contact rates, the main outcomes were obtained by running the simulation for each of three scenarios: **(A)** without contact investigations, **(B)** with contact investigations and less intervention delays for contacts of the index case, and **(C)** with contact investigations and more intervention delays for contacts of the index case. For scenarios A, B, and C, red cells show the combinations with uncontrolled outbreaks and blue cells represent the simulated outbreak sizes of the combinations without uncontrolled outbreaks. The values of simulated uncontrolled outbreak probabilities are shown with blue numbers in red cells; the values of simulated outbreak sizes are shown with red numbers in blue cells. The frontiers between adjacent combinations with and without uncontrolled outbreaks are shown by the black lines. These simulations suggest: (1) contact investigations play an important role in preventing uncontrolled measles outbreaks and reducing the total outbreak size; (2) without contact investigations but with the lowest scale of contact rates in all places, even an 80% vaccination coverage may prevent uncontrolled measles outbreaks; (3) with contact investigations, reducing the contact rates in all settings may lower the vaccination coverage required to prevent uncontrolled measles outbreaks; (4) with contact investigations and the highest vaccination coverage level, uncontrolled measles outbreaks may be prevented even with very high contact rates in all settings; (5) less intervention delay for contacts of the index case may help contact investigations reduce the number of measles cases.

To study further the effects of contact rates in all places and vaccination coverage on the uncontrolled measles outbreak probability and outbreak size, we used a parameter ranging from 0 and 1 to scale contact rates in school, daycare, workplace, household and neighborhood settings (i.e., when the scale parameter is 0, each place has the lowest contact rate; when the scale parameter is 1, each place has the highest contact rate; when the scale parameter is between 0 and 1, the contact rate in each place is between its lower and upper bounds shown in Table 1). Similarly, for each combination of vaccination coverage (ranging from 0 to 1) and the scale parameter (ranging from 0 to 1), we ran the simulations under three scenarios (all other the parameters’ values are shown in Table 1): (1) without contact investigation, (2) with contact investigation and less intervention delay for contacts of the index case, and (3) with contact investigation and more intervention delay for contacts of the index case. For the scenario without contact investigation, Figure 4(A) shows that the more contact rates in all places the more outbreaks and the higher number of outbreak sizes; increasing vaccination coverage could reduce the number of measles cases; when scale of contact rates in all places was very high, even the highest vaccination coverage (100%) could not completely prevent the measles outbreak; however, for the lowest scale of contact rates, even 80% of vaccination could prevent measles outbreak. For the scenario with contact investigation and less intervention delay for contacts of the index case, Figure 4(B) shows that with lower scale of contact rates in all places, measles could be controlled even with 80% of vaccination coverage; and 100% of vaccination coverage could prevent measles outbreak for all scales of contact rates in all places. For the scenario with contact investigations but with more intervention delays among contacts of the index case, Figure 4(C) shows that measles outbreaks were prevented by the combinations of low contact rates in all places and all vaccination coverage levels, and the combinations of the highest vaccination coverage and all contact rates in all places; most of the uncontrolled outbreak probabilities and the outbreak sizes were higher than those of the scenario with contact investigations and less intervention delays for contacts of the index case (in Figure 4(B)) because the effects of intervention delays. By comparing this to the same combinations of the scenario without contact investigations in Figure 4(A), the probabilities of an uncontrolled outbreak and the outbreak sizes in the other two scenarios in Figures 4(B) and (C) were dramatically reduced by the contact investigations. These simulations suggest: (1) contact investigations play an important role in preventing measles outbreaks and reducing the total outbreak sizes; (2) without contact investigations but with the lowest scale of contact rates in all places, even an 80% vaccination coverage may prevent measles outbreaks; (3) with contact investigations, reducing the contact rates in all places may lower the vaccination coverage required to prevent measles outbreaks; (4) with contact investigations and the highest vaccination coverage level, measles outbreaks may be prevented even with very high contact rates in all places; (5) less intervention delays for contacts of the index case may help contact investigations reduce the number of measles cases.

## Discussion and conclusion

We constructed a stochastic agent-based model to explore the impact of various characteristics (population vaccination coverage, clustering of immunity, individual behavior, and the public health response) on the occurrence of measles epidemics. Our analysis focused on contact investigations and their associated interventions (post-exposure prophylaxis) to control and prevent measles transmission. Our results confirm previous observations that vaccination coverage and clustering of immunity are important characteristics that influence the ability to control a measles epidemic. We were able to expand on our understanding of vaccination coverage and clustering by demonstrating that the prevention of uncontrolled measles outbreaks may depend on the level of intervention delays and the probability of finding a contact when vaccination coverage is high with a low to moderate level of clustering. In a highly immunity-clustered population, the prevention of uncontrolled measles outbreaks may only be possible with high vaccination coverage levels (e.g., 95% vaccination coverage). Furthermore, we showed that contact rates are important drivers of transmission, especially neighborhood contact rates given our assumptions about our ability to identify contacted individuals in the neighborhood setting. Reducing contact rates in all settings may lower the vaccination coverage level required to prevent measles outbreaks in the presence of contact investigations and their associated interventions. Overall, the results demonstrated the importance of contact tracing activities to prevent uncontrolled measles outbreaks or reduce the total outbreak size.

Previous mathematical epidemiology studies have examined the factors that are needed to sustain measles transmission in communities [44-46], including critical community size [47-49] and herd immunity [50,51]. In addition, the effects of contact patterns across network structures, including clustered and complex networks, have also been elucidated using mathematical models in order to design effective control strategies [8,9,13,52-55]. While there have been studies that have examined the role of contact tracing to control infectious diseases among clustered populations [56-58], none have modeled the public health response using an agent-based model as we have done in this study. The agent-based model created using an open-source software system (FRED) allows refinements to the model to be made for further explorations of the effects of the public health response, individual behaviors, population vaccination coverage, and network structures on the ability to control and prevent measles outbreaks.

However, the study is subject to several limitations. First, we do not have a lot of empirical evidence for many of the behavior-related parameters in our model. We obtained, where possible, expert opinion and/or published estimates from the literature to inform our parameter values. We incorporated a broad range of values for the behavioral-related parameters in order to explore the wide range of outcomes and conducted a sensitivity analysis of the simulation results. Second, we maintained a balance between core individual behaviors and public health response behaviors in our model with specific nuanced scenarios or possibilities. For example, we understand that children ages 6 months to 11 months can be given one dose of MMR vaccine instead of intramuscular IG if it is given within 72 hours of exposure so long as the child is vaccinated again after 12 months of age; our stochastic agent-based model does not allow for this possibility with this age group. Third, we only considered vaccination clustering status among children less than 18 years of age. Clustering among children occur because parents make decisions for their children regarding vaccination. However, there are little data on vaccination clustering among adults. Finally, the contact network of this model did not include the healthcare settings where measles cases interact with healthcare workers and healthcare workers who become cases and work while infectious may have a large number of contacts.

Our results support the use of contact investigations in the control and prevention of measles epidemics; the results elucidate the circumstances in which control is possible for various levels of vaccination coverage and clustering of susceptibility among households, and the simulation results are consistent with the findings in [59] in which the authors found that minimal changes in the level of aggregation of unvaccinated individuals can lead to sustained transmission in highly vaccinated populations.

We used an agent-based model to simulate measles transmission after the introduction of one index case into a population and study the effects of the level of immunity clustering, vaccination coverage, contact rates, contact tracing and public health interventions on the uncontrolled measles outbreak probability and the total outbreak size in 365 days. The simulated results show that the level of immunity clustering, vaccination coverage and contact investigation are important in the prevention of measles outbreaks. With contact investigations and moderate contact rates in all places, measles outbreaks can be prevented by (1) a very high vaccination coverage (≥95%) with a moderate to low level of immunity clustering (≤0.5), or (2) a moderate vaccination coverage (85% or 90%) with the lowest level of immunity clustering and a short intervention delay for contacts of the index case and a high probability that a contact can be traced. Without contact investigations, measles outbreaks may be prevented by the highest vaccination coverage with the lowest level of immunity clustering and moderate contact rates. For the highest contact rates in all places, even the lowest level of immunity clustering with the highest vaccination coverage cannot completely prevent measles outbreaks. Two directions for future work include using this model (with calibration) to study: (1) the effects of public health intervention strategies on the control and prevention of measles epidemics, and (2) the effects of clustering of susceptibility in school and daycare settings on vaccination coverage required to prevent measles outbreaks.

## Additional file

Additional file 1:
**Appendix for “The role of vaccination coverage, individual behaviors, and the public health response in the control of measles epidemics:** an agent-based simulation”.
