# Supplementary material for: The role of vaccination coverage, individual behaviors, and the public health response in the control of measles epidemics: an agent-based simulation for California
Source: BMC Public Health. 2015 May 1;15:447. doi: 10.1186/s12889-015-1766-6 (PMC4438575; doi:10.1186/s12889-015-1766-6)
Supplement: Additional file 1: — Appendix for “The role of vaccination coverage, individual behaviors, and the public health response in the control of measles epidemics: an agent-based simulation”. [file 12889_2015_1766_MOESM1_ESM.pdf]

Appendix for “The role of vaccination coverage, individual behaviors, and the public health response in the control of measles epidemics: an agent-based simulation for California”

1 Introduction

In this Appendix, we provide full details about the model structure and parameters we used to simulate contact investigations and public health interventions for the control and prevention of measles transmission.

2 Formal Specification

Individual-based modeling is a relatively new approach to modeling complex systems composed of interacting, autonomous individuals [1]. Individuals have behaviors, often described by simple rules, and interactions with other individuals, which in turn influence their behaviors. By modeling individuals, the full effects of the diversity can be observed as it gives rise to the behaviors of the system as a whole. A typical individual-based model often has three basic elements: 1) a set of individuals that includes attributes and behaviors; 2) a set of individual relationships and methods of interaction, which define how and with whom individuals interact; 3) an environment where an individual obtains more information to adjust his/her attributes and behaviors [1-3].

We describe the specification of the discrete-time, discrete-event system which constitutes our model (using the terminology and theory of [4]). Thus, we specify five components: (1) the state space of the model, (2) the set of possible events, (3) the set of active events of each possible state, (4) the transition function which specifies how the state of the system changes when an event occurs, and finally (5) the parameter values.

Our model can be formalized as a generalized semi-Markov scheme (GSMS) of a discrete-event system [4]. In a system of a population with *N* individuals, we assume that there are *k* state types for each individual with a set of state types, $Y = \{Y_1, Y_2, \cdots, Y_k\}$. For example, the set of state types for individual *j* can be expressed by $Y_j = \{Y_{j,1}, Y_{j,2}, \cdots, Y_{j,k}\}$. Then we have the state space (Cartesian product) of individual *j*, $X_j = Y_{j,1} \times Y_{j,2} \times \cdots \times Y_{j,k} = \{X_j^{(1)}, X_j^{(2)}, \cdots, X_j^{(m)}\}$, where $m = \prod_{i=1}^{k} |Y_{j,i}|$, and each $x \in X_j$ is a specific state of individual *j*. Therefore, the state of the population as a whole system can be represented by the states of all of the individuals. We assume S as the state space of the population, then $S = X_1 \times X_2 \times \cdots \times X_j \times \cdots \times X_N = \{s_1, s_2, \cdots, s_n\}$, where $n = \prod_{j=1}^{N} |X_j|$, and each $s \in S$ is a unique state of the population. For each $s \in S$, there is a set of active events ($E(s)$), each active event $e \in E(s)$ can change the states of one or more individuals in the population with state s. With the action of event e, state s could move to another different state s' with probability p, $p(s'; s, e)$. A universal event A, $\mathrm{A} = \bigcup_{s \in S} E(s)$, is an event set for S. According to the definition of GSMS in [4], the population state space S and event set A (as we described above) construct a GSMS. In this population system, when we specify an initial state s^0, we assume that all other

states in S are reachable from s^0 as follows: (1) for every $s \in S$, there exist states $s^1, \cdots, s^r$ and events $e^0, e^1, \cdots, e^r$ with $e^i \in E(s^i)$, $i = 0,1,\cdots,r$, and (2) $p(s^1; s^0, e^0) p(s^2; s^1, e^1) \cdots p(s; s^r, e^r) > 0$; any state in this population can be reached by other states by changing some individuals' states.s

This population system of GSMS is a generalized semi-Markov process (GSMP) during the simulation. Each active event has an event time associated with it; the simulation proceeds by updating the current time from *t*-1 to *t* (the time unit in the simulation is one day) and choosing the active events which event times are *t*. The state of the system is then transformed according to the chosen events [4], and this simulation time, *t,* is updated to the time *t*+1. This process yields a sequence of event occurrence times $t^0, \cdots, t^q, \cdots$ (for $0 \le i \le q$, t^i and t^{i+1} may be the same, which means different active events may have the same event time but may be ordered according their priorities), and a corresponding sequence of the state values at these times $s^0, \cdots, s^q \cdots$, for each event time index $q = 0,1,\cdots$. Thus, s^0 is the initial condition of the system at time t^0; the first event occurs at time t^1 and causes the state of the system to change from s^0 to s^1, and so forth. When such a state change takes place, the system will choose active events based on the updated state. We first specify the state S of the system, and then each of the possible events.

3 State Specifications

3.1 State of Individuals

We first specify the states of the individuals in the model. Individuals are indexed by *j*, *j* = 1,2, …, *N*(*t*), where *N* is the size of the simulated population on day *t* (the time unit in the model is one day). For individual *j*, some important individual states (see the Table 1) on day *t* include: (1) the current stage of measles, G_j (*t*); (2) the start date of exposure, E_j; (3) the start date of infectious, I_j; (4) the start date of symptoms, Sym_j; (5) the start date of recovery, R_j; (6) the start date public health intervention, V_j (*t*); (7) the current protection by previous MMR vaccinations, P_j (*t*); (8) the current age, A_j (*t*); (9) the current household location for individual *j*, H_j (*t*); (10) the current school location for individual *j*, Sch_j (*t*); (11) the current daycare location for individual *j*, $Daycare_j$ (*t*); and (12) the current workplace location for individual *j*, W_j (*t*). The states for individual *j* on the next day, *t=t+1*, will be updated based on its current states, the system global variables and interactions with others on day *t*. The system global variables and the interactions between individuals will be discussed in Sections 3.2 and 4.2.

Stage of Measles. Assuming that *t* is the current day, an individual can be in a stage G_j of Susceptible (S), Exposed (E), Infectious (I) or Recovered (R). It is convenient to define $G_j(t)$ as an indicator function (which will be stated in the section of Events/Functions) of whether individual *j* is susceptible, exposed, infectious or recovered on current day *t*, i.e.,

$$G_j(t) = \begin{cases} S, t < E_j \ or \ E_j = -1 \ or \ I_j = -1 \ or \ R_j = -1 \\ E, E_j \le t < I_j \\ I, I_j \le t < R_j \\ R, t \ge R_j \end{cases}$$

, where E_j, I_j, and R_j are the start dates of exposure, infectiousness and recovery respectively. Once individual *j* is infected, the start date of exposure is recorded, and the start dates of infectiousness and recovery are randomly generated from the observed distributions of latent period and symptomatic period as shown in Table 3.

Start Date of Exposure. For individual *j*, he or she may have contact with many other individuals in his or her social settings (for the contact network and the process of transmission per contact, please see Section Events/Functions: Transmission). If there was an infectious contact on day t_E (several days before current day t) and individual j was infected, then t_E would be the start date of exposure, i.e., $E_j = t_E$. The default date of exposure is -1 which indicates the individual has never been infected by measles since the beginning of the simulation.

Start Date of Infectiousness. When individual *j* is exposed and infected by someone on day E_j, a projection of latent period will be randomly generated from the observed distribution of the latent period for measles. Assuming a randomly generated latent period is t_{latent_r}, the first day individual j becomes infectious will be scheduled on t_{latent_r} days after exposure date E_j, that is $I_j = E_j + t_{latent\_r}$. The infectious start date may be rescheduled or even canceled if individual *j* is traced and given interventions by public health workers. The intervention measures include post exposure prophylaxis (PEP) (MMR vaccine within 72 hours after the exposure date or PEP immune globulin (IG) within 6 days after the exposure date [5] -- please see section Events/Functions: Public Health Contact Tracing & Interventions for further details).

Start Date of Symptoms. Similarly, for individual *j* who has been exposed and in an infection course of measles, the start date of symptoms is defined as

$$Sym_j = E_j + t_{incubation\_r},$$

where $t_{incubation\_r}$ is a random incubation period generated from the cumulative probability distribution of measles incubation period according Table 3. For simplicity we assumed that the latent period and the incubation period for measles are identical. The symptomatic date may be rescheduled or even canceled if individual *j* is traced by public health workers and is given

certain interventions, such as PEP MMR vaccine within 72 hours after exposed date, or PEP IG within 6 days after exposure date.

Start date of Recovery. For individual *j* who has been in an infection course, the start date of recovery will be scheduled by predicting the duration of infectiousness. The infectious period for individual *j* can be randomly generated from the observed distribution of measles infectious duration; therefore, the start date of recovery, R_j , may be several days after the start date of infectiousness, i.e.,

$R_j = I_j + t_{infectiousness\_r}$,

where $t_{infectiousness\_r}$ is a random infectious period. Again, the recovered date for individual *j* may be rescheduled to an earlier date if public health personnel find the case quickly and give the case an effective intervention of either PEP MMR or PEP IG (within 72 hours or 6 days of the exposure date respectively). Please see section Events/Functions: Public Health Contact Tracing & Interventions for details.

Start Date of Public Health Intervention. If the indicators (global variables θ_{20} and θ_{21} in the Table 2) of Public Health Contact Tracing and Public Health Intervention are active, individual *j* , who has been exposed to an index case *i* on day E_j and started the infection course, can be found by public health workers by tracing from individual *i*. There is a delay between the exposure date of individual *j* and the identified date of individual *j*, because the index case *i* may need some time t_{delay_report} (the time from the infectious start data I_i to the date his/her symptoms become obvious) to report his/her symptoms to a doctor (assuming index case *i* had not reported on day E_j), and public health workers may also need response time ($t_{delay\_trace\_case}$) to speak with the identified index case *i* and trace to his/her contact individual *j*. Therefore, the start date of the public health intervention for individual *j* (a traced case), V_j, can

be defined as:

$V_j = E_i + t_{delay\_report} + t_{delay\_trace\_case}$,

i.e., several days (the response time) after the date when the index case *i* reports his/her symptoms. Similarly the start date of the public health intervention for a self reported case *i*, V_i can be defined as

$V_i = E_i + t_{delay\_report} + t_{delay\_reported\_case}$,

where $t_{delay\_reported\_case}$ is the response time for an index case or reported case, which should be faster than the response time for a traced case like individual *j*. The times t_{delay_report} , $t_{delay\_trace\_case}$ and $t_{delay\_reported\_case}$ are global variables denoted by θ_3, θ_4 and θ_5 respectively in Table 2. There are two other variables needed for the public health intervention: the duration of the intervention and the end date of the intervention. Because the PEP MMR and PEP IG interventions can be given in one day, we assume the duration is 0 and the start date is the same as the end date. The Home Quarantine may last several days until the date of recovery, so the duration for home quarantine begins with the start date of the intervention and ends with the start date of recovery.

Immunity from Previous MMR Vaccinations. When individual *j* comes in contact with an infectious measles case, he or she may not become infected because of his or her immunity status. We assume that immunity is from previous MMR vaccination. If individual *j* has been vaccinated before the current day *t*, then the immunity protection, P_j, is same as the MMR effectiveness (Table). The default value for P_j is zero which indicates that this individual has no immunity for measles. For details how immunity is generated and how it protects individuals from infection, please refer to the Initialization and section Events/Functions: Transmission section.

Age. The age of individual j, A_j, can be increased on his or her birthday (each individual is initialized with an age and birthday) every year, and age can be used to determine the chance the individual will be covered by mass MMR vaccine (the coverage is age dependent), and to decide which intervention is suitable for the individual (e.g., PEP MMR is not routinely given to individuals less than 12 months of age [5]).

Household Location. Each individual must have information about household location. For individual j, the household location, H_j, is a vector with three elements, H_j = (identification number (ID), longitude, and latitude), which can be used to include j's family members into the same household, and create a contact network between the household and a contact network in neighborhood (please see FRED user's guide [6, 7], RTI's synthetic population [8], and section Events/Functions: Transmission for details).

School Location. School-aged individuals may have the school location information, Sch_j = (ID, longitude, and latitude), which is used to generate the contact networks of schools. If individual j is not a school student, the school ID, longitude and latitude are -1.

Daycare Location. Daycare-aged individuals may have the daycare location information, $Daycare_j$=(ID, longitude, latitude), which is used to generate the contact networks of daycares. If individual j is not in a daycare, the daycare ID, longitude and latitude are -1. To generate the synthetic daycare data, we assumed that each daycare-aged child has a daycare ID with some probability and assigned the daycare as follows: (1) choose the two closest available daycares to the workplaces of the child's parents (if either parent doesn't have a workplace, the selected daycare ID is -1); (2) choose the closest available daycare to the child's house; (3) from the daycare(s) selected by steps (1) and (2), pick a daycare which has the shortest distance to the child's house or the workplaces of the child's parents.

Workplace Location. Similarly, W_j = (ID, longitude, and latitude) is the workplace location for individual j. The location information and the age and gender of the individual are based on the synthetic California population data created by RTI and synthesized using the U.S. census and the California Department of Finance population estimates.

3.2 Global Variables

Global variables are defined in Table 2; we use notations θ_1 to θ_{32} to represent each of the global variables, and use θ to represent the collection of global variables. The global variables fall into the following categories: behavior variables (θ_1, θ_2, θ_7, θ_{29}), infection variables (θ_5, θ_{22}, θ_{23}, θ_{25}, θ_{26}, θ_{27}, θ_{28}, θ_{31}), social network variables (θ_{14}, θ_{15}, θ_{16}, θ_{17}, θ_{18}, θ_{20}, θ_{30}), contact tracing variables (θ_6, θ_{11}, θ_{12},), and intervention variables (θ_3, θ_4, θ_8, θ_9, θ_{10}, θ_{13}, θ_{19}, θ_{21}). Please see Table 2 for a detailed interpretation of each variable.

3.3 Full State Space

The full state space $\boldsymbol{X}_j$ for an individual in the system is specified by the collection

$\boldsymbol{X}_j = \{ G_j, E_j, I_j, Sym_j, R_j, P_j, A_j, V_j, H_j, Sch_j, Daycare_j, W_j, \theta\}$,

where G_j denotes the collection of measles stage values for each individual j, etc. The full state space $\boldsymbol{S}$ for the population system is specified by the collection of each individual's state space:

$S = X_1 \times X_2 \times \cdots \times X_j \times \cdots \times X_N = \{s_1, s_2, \cdots, s_n\}$, where $n = \prod_{j=1}^{N} |X_j|$, and each $s \in S$ is a unique state of the population.

3.4 Initial Conditions

The model is initialized with $N(0)$ individuals, the initial population size is equal to the population of a county in California. For each individual j, its age (A_j), household location (H_j),

school location (Sch_j), and workplaces location (W_j) were generated based on the census data by RTI. The daycare locations were obtained from the California Department of Social Services' Community Care Licensing Division website (http://www.ccld.ca.gov). With a probability that parents are willing to send their child to a daycare, we assigned each daycare-age child *j* an available daycare ID, Daycare_j, which has the smallest distance to the child's household or to one of the child's parents' workplaces.

We assume that measles vaccination had been given (θ_{19}=TRUE) to the population before the initial date (t=0), and vaccine protection for individual *j* is θ_{12} if it had been covered with by vaccination, i.e., $P_j = \theta_{12} * \delta_R(vac_{Coverage}(A_j))$, where $vac_{Coverage}(A_j)$ is the probability that an individual at age A_j is covered by vaccine (please see Table 5 for the age-dependent vaccination coverage; for children whose age <=18, instead of using the age-dependent vaccination coverage, we used a variable of coverage between 0.85 to 1 and a variable of clustering which is a household-level vaccination acceptance rate between 0 and 1, i.e., given a vaccination coverage and a clustering level, each child <= 18 in a household is covered by vaccination with the vaccination coverage if and if only the household is willing to accept vaccination with 1 – the value of clustering level), *R* is a random number between 0 and 1 from a uniform distribution (hereafter the same), and

$$\delta_R(vac_{Coverage}(A_j)) = \begin{cases} 1, R \le vac_{Coverage}(A_j) \\ 0, R > vac_{Coverage}(A_j) \end{cases}.$$

We also assume that there is only one index measles case (θ_{23}=1) on the first day of the simulation. To choose an individual as an initial case: we (1) randomly choose an individual according to the age distribution of measles cases in California; and (2) accept the individual as an index case if his/her age-dependent immunity is not enough for protection from measles

infection, using a function

$$\delta_R(\theta_{12}) = \begin{cases} 1, R \leq \theta_{12} \\ 0, R > \theta_{12} \end{cases};$$

(3) if the person is not accepted as an index case then steps (1) and (2) are repeated until an individual has been accepted as the index case. For index case *i*, its exposure date is the first day (E_i=0); its start date of infectiousness $I_i = E_i + t_{latent\_r}$, where t_{latent_r} is randomly generated from the distribution of observed latent period of measles; and its start dates of symptoms ($Sym_i = E_i + t_{incubation\_r}$) and recovery ($R_i = I_i + t_{infectiousness\_r}$) are also generated based on a random incubation period $t_{incubation\_r}$, and a random infectiousness period $t_{infectiousness\_r}$.

As a base case, the *Public Contact Tracing* (θ_{20}=TRUE) and the *Public Health Intervention* (θ_{21}=TRUE) are assumed to be activated. Therefore, the start date of public health intervention for the index case *i* will be scheduled on day $V_i = Sym_i + t_{delay\_report} + t_{delay\_reported\_case}$, where the value of t_{delay_report} depends on parameter θ_5, and the value of $t_{delay\_reported\_case}$ depends on parameter θ_3. The index case will become infectious on day I_i with a maximum infectivity (θ_{22}=0.99), and spread measles to others via its daily contacts in household, neighborhood, school, daycare and workplace.

To control and prevent possible epidemics caused by the index case, all three interventions (PEP Vaccine, PEP IG and Home Quarantine) are enabled (θ_8=(1,1,1)). The effectiveness is θ_{10} for PEP MMR Vaccine, and θ_9 for PEP IG. The probability that an individual complies with the Home Quarantine is θ_1. The cooperative probability for an individual to accept an intervention is θ_7. Once the index case reports itself, public health workers have a chance (θ_2) to get a contact name (id) from the identified case, and have chance (θ_6) to find out the contact the index case contacted with. We assume that public health workers are interested to find all of the contacts of

index case i in the past θ_{11} days (from the day $E_i + t_{delay\_report}$ back to the day $max(0, E_i + t_{delay\_report} - \theta_{11})$), in the places indicated by parameter θ_{12}=(Household, School, Daycare and Workplace).

The contact networks (social network) of this population system consist of five types: Household, School, Daycare, Workplace, and Neighborhood. They are initialized as follows.

All individuals are indexed by their Household IDs, assuming there are total $N_{household}$ (≥ 1) unique Household IDs represented by $\{h\_id_u\}$ where u = 1, 2, …, $N_{household}$, then one household h_u is a collection of individuals with the same household ID h_id_u, i.e.

$h_u=\{ j : H_j(\text{id}) = h\_id_u\}$

where j is the index of individual and u is the index of Household ID. Therefore, all households can be defined as a set

$Households=\{h_u\}$, u = 1, 2, …, $N_{household}$.

To find the household contacts of individual j, we can first compare its household id H_j(id) to $\{h\_id_u\}$ and locate to the uth element that $h\_id_u = H_j(\text{id})$, then the uth element in $\{h_u\}$, h_u, is the household member collection in which all individuals have the same household id and location as individual j. For each household member in h_u, individual j may contact a household member every day with a probability θ_{14}.

Similarly the contact networks of School, Daycare and Workplace can be constructed by using the method stated above.

Based on individuals' household locations (longitude and latitude), a neighborhood is assumed to be on a grid with 1 km^2. Assuming that all individuals are located in an area with total $N_{neighborhood}$ 1 km^2 grids represented by $\{ nbhd\_cell_i \}$ where i is 1, 2, …, $N_{neighborhood}$, then one

neighborhood $nbhd_i$ is a collection of individuals whose households are located in the same grid $nbhd\_cell_i$, i.e.,

$nbhd_i$={ j : H_j(longitude)∈[$nbhd\_cell_i$(longitude$_{min}$), $nbhd\_cell_i$(longitude$_{max}$)] and H_j(latitude)∈ [$nbhd\_cell_i$(latitude$_{min}$), $nbhd\_cell_i$(latitude$_{max}$)]}

where *j* is the index of individual and *i* is the index of cell. All neighborhoods can be defined as a set

Neighborhoods={$nbhd_i$}, *i* = 1, 2, …, $N_{neighborhood}$.

Individual *j*'s home neighborhood is the grid in which its household is located. To find the home neighborhood contacts of individual *j*, we can compare its household location H_j(longitude, latitude) to {$nbhd\_cell_i$} and locate to the *i*th element that $H_j(longitude)$∈$[nbhd\_cell_i(longitude_{min})$, $nbhd\_cell_i(longitude_{max})]$ *and* $H_j(latitude)$∈$[nbhd\_cell_i(latitude_{min})$, $nbhd\_cell_i(latitude_{max})]$, then the *i*th element in {$nbhd_i$}, $nbhd_i$, is the sleeted neighborhood in which all individuals' households are in the same grid. However, individual *j* may visit another neighborhood in the community during a given day. The decision about where to spend the neighborhood activity period is made independently each day, with the highest probability to visit the home neighborhood, and a lesser probability to visit one of the surrounding neighborhoods, and a small probability of visiting a randomly selected neighborhood within a given community radius with θ_{18} km. On each simulated day, individual *j* randomly contacts some of neighborhood members in $nbhd_i$, with a daily contact rate θ_{17}.

The end day of the simulation, θ_{24}, is 365.

The epidemic is initiated by randomly selecting an initial case on day *t*=0, and letting the initial case contact others during the period of infectiousness. With the initial state $s^0=(X_1(0),\ldots,X_N(0))$, the population system will choose active events on day *t*=0 for each

individual *j* based on its state $X_j(0)$, and update each individual's state to $X_j(1)$, then update system state to s^1 on day t=1; repeating this transition process until the end day $t=\theta_{24}$ will get system state s^t (t = 0, 1, …, θ_{24}) on each day.

4 Events/Functions

A set of important events is listed in Table 3. The event functions, active conditions and details will be discussed in turn. Any individual state variables (components of state space $\boldsymbol{X}_j$) whose values are not specified for any particular event are assumed to remain unchanged.

4.1 Health Update

Function. This is an individual-level function which updates the current day's measles stage for a given individual according to its scheduled start date of exposure, infectiousness and recovery. This function also calls another individual level function, ***Public Health Intervention,*** once the current day is the scheduled start date of the public health intervention for the given individual.

Input parameters. Current day *t,* Current state for individual *j*, $X_j(t) = (G_j(t), E_j(t), I_j(t), Sym_j(t), R_j(t), P_j(t), A_j(t), V_j(t), H_j(\mathrm{t}), Sch_j(t), Daycare_j(t), W_j(t), \theta)$.

Conditions. This function is active for individual *j*, if and only if (iff), the current day *t* is the start date of exposure $t=E_j$, OR is the start date of infectiousness $t=I_j$, OR the start date of public health intervention $t=P_j$, OR the start of recovery $t=V_j$. If any of these conditions is satisfied by individual's current state $X_j(t)$, then *Health Update* is active and updates individual *j*'s state for the current day as follows.

Details. It updates measles stage by $G_j(t) = \begin{cases} S, t < E_j \text{ or } E_j = -1 \text{ or } I_j = -1 \text{ or } R_j = -1 \\ E, E_j \le t < I_j \\ I, I_j \le t < R_j \\ R, t \ge R_j \end{cases}$.

It calls function ***Public Health Intervention***, if t=P_j, to choose the intervention method for individual *j*. If current day is the recovery date t=$R_j(t)$ and individual *j* has been tagged with ***stay home***=TRUE, then set the tag ***stay home***=FALSE indicating that individual *j* is not restricted by Home Qurantine. Other individual variables are not changed by this function. The new state of individual *j* on the current day *t* is *Health Update*($X_j(t)$) = $\boldsymbol{G_j(t)}$.

4.2 Transmission

Function. This is an individual-level function which takes the current states of two individuals (infectious individual *i* and susceptible individual *j*) along with other inputs, and tries the transmission of measles from *i* to *j*. If it is a successful transmission, the measles stage for individual *j* will be updated from susceptible to exposed, and another function ***Trajectory*** will be called to schedule the start dates of infectiousness, symptoms and recovery for the newly-infected individual *j*. A function ***Contact History List Update*** will be called to record when and where the infectious case *i* contacted the susceptible *j*.

Input parameters. Current day *t*; Current state for infectious individual *i*, $X_i(t)$ = ($G_i(t)$, $E_i(t)$, $I_i(t)$, $Sym_i(t)$, $R_i(t)$, $P_i(t)$, $A_i(t)$, $V_i(t)$, H_i(t), $Sch_i(t)$, $Daycare_i(t)$, $W_i(t)$, θ); Current state for susceptible individual *j*, $X_j(t)$ = ($G_j(t)$, $E_j(t)$, $I_j(t)$, $Sym_j(t)$, $R_j(t)$, $P_j(t)$, $A_j(t)$, $V_j(t)$, H_j(t), $Sch_j(t)$, $Daycare_j(t)$, $W_j(t)$, θ); Contact place where the infectious *i* contacts *j*: $\text{contact}_{\text{place}}(i, j)$ =

Household, Neighborhood, School, Daycare or Workplace; Transmission probability per contact in (Household, School, Workplace, Neighborhood, Daycare) = (θ_{25}, θ_{26}, θ_{27}, θ_{28} θ_{31}); Infectivity of infectious case: θ_{22}.

Conditions. This function is active if and only if the individual *i* is infectious and individual *j* is susceptible on the current day: $G_i(t)$=*Infectious* AND G_i(t)=*Susceptible*. If these conditions are satisfied by the current state of infectious individual $X_i(t)$ and the current state of susceptible individual $X_j(t)$, then *Transmission* is active and updates infectious individual *i*'s state and susceptible individual *j*'s state to new states for the current day.

Details. When *i* contacts *j*, the probability of successful transmission (i.e., susceptible individual *j* is infected by infectious individual *i*) trans_prob(*i*, *j*) is calculated by

trans_prob(*i*, *j*) = trans_prob_contact(*i*, *j*) * infectivity(*i*) * susceptibility(*j*),

where trans_prob(*i*, *j*) is the transmission probability per contact, infectivity(*i*) is the level of infectivity between 0 and 1 indicated by parameter θ_{22}, the susceptibility(*j*) is the chance that *j*'s previous immunity cannot protect *j* from this infectious contact, and the value of the chance is estimated by 1-V_j. In addition, trans_prob(*i*, *j*) depends on the place where *i* contacts *j*, $contact_{place}(i, j)$:

$$\text{trans\_prob\_contact}(i, j) = \begin{cases} \theta_{25}, contact_{place}(i,j) = Household \\ \theta_{26}, contact_{place}(i,j) = School \\ \theta_{27}, contact_{place}(i,j) = Workplace \\ \theta_{28}, contact_{place}(i,j) = Neighborhood \\ \theta_{32}, contact_{place}(i,j) = Daycare \end{cases}.$$

Then the successful transmission probability from *i* to *j* is:

trans_prob(*i*, *j*) = trans_prob_contact(*i*, *j*) * θ_{22} * (1-V_j).   Giving a random number R between 0

and 1, j is decided to be infected or not by this function:

δ_R(trans_prob(i, j)) = $\begin{cases} 1, R \le trans\_prob \quad (i,j) \\ 0, R > trans\_prob \quad (i,j) \end{cases}$.

If δ_R(trans_prob(i, j)) is 1: the start date of exposure for individual j will be adjusted to be the current day $E_j(t)$= $\begin{cases} t, \delta_R(trans\_prob\ (i,j)) = 1 \\ E_j(t), \delta_R(trans\_prob\ (i,j)) = 0 \end{cases}$; and the measles stage for individual j will be updated from susceptible to exposed $G_j(t)$= $\begin{cases} E, \delta_R(trans\_prob\ (i,j)) = 1 \\ G_j(t), \delta_R(trans\_prob\ (i,j)) = 0 \end{cases}$; and another function ***Trajectory*** will be called to schedule its start dates of infectiousness, symptoms and recovery. The ***Contact History List Update*** will be called (regardless transmission is successful or not) to record when and where the infectious case i contacted the susceptible j.

Other individual variables for j are unchanged, and we have j's new state for the current day by ***Transmission***$\boldsymbol{(X_i(t), X_j(t))}$ = ($\boldsymbol{G_j(t), E_j(t)}$). Infectious individual i's variables are unchanged by event *Transmission*.

4.3 Trajectory

Function. This function works on individual level. It schedules the start dates of infectiousness, symptoms and recovery for a new infected individual.

Input parameters. Current day t; Current state for a new infected individual j, $X_j(t)$ = ($G_j(t)$, $E_j(t)$, $I_j(t)$, $Sym_j(t)$, $R_j(t)$, $P_j(t)$, $A_j(t)$, $V_j(t)$, H_j(t), $Sch_j(t)$, $Daycare_j(t)$ $W_j(t)$, θ); Incubation period distribution: cdf$_{incubation}$; Latent period distribution: cdf$_{latent}$; Infectious period distribution: cdf$_{infectiousness}$.

Conditions. This function is active if and only if the new infected individual *j* is exposed on the current day: $G_j(t)$=*Exposed* AND $t= E_j(t)$.

Details. For the new infected individual *j*, *j*'s exposed start date has been updated to the current day, $E_j(t)=t$, by *Transmission*. Individual *j*'s start date of infectiousness, $I_j(\mathrm{t}) = E_j(t) + t_{latent\_r}$ is randomly generated from the distribution of observed latent period for measles cdf_{latent}. The new infected *j*'s start dates of symptoms ($Sym_j(t) = E_j(t) + t_{incubation\_r}$) and recovery ($R_j(t)=I_j(t) + t_{infectiousness\_r}$) can be randomly generated from the distributions of incubation period $\mathrm{cdf}_{incubation}$ and infectiousness period $\mathrm{cdf}_{infectiousness}$.

Other variables for *j* are unchanged, the new infected *j*'s state for the current day *t* is updated by $Trajectory(X_j(t))$ = ($\boldsymbol{I_j(t)}$, $\boldsymbol{Sym_j(t)}$, $\boldsymbol{R_j(t)}$).

4.4 Contact History List Update

Function. This function works on individual level. It records where and when an infectious individual *i* contacted a susceptible individual *j*.

Input parameters. Current day *t*; Current state for infectious individual *i*, $X_i(t)$ = ($G_i(t)$, $E_i(t)$, $I_i(t)$, $Sym_i(t)$, $R_i(t)$, $P_i(t)$, $A_i(t)$, $V_i(t)$, $H_i(\mathrm{t})$, $Sch_i(t)$, $Daycare_i(t)$, $W_i(t)$, θ); Current state for susceptible individual *j*, $X_j(t)$ = ($G_j(t)$, $E_j(t)$, $I_j(t)$, $Sym_j(t)$, $R_j(t)$, $P_j(t)$, $A_j(t)$, $V_j(t)$, $H_j(\mathrm{t})$, $Sch_j(t)$, $Daycare_j(t)$, $W_j(t)$, θ); Contact place where the infectious *i* contacts *j*: $\mathrm{contact}_{\mathrm{place}}(i, j)$ = Household, Neighborhood, School or Workplace; Days of tracing back: θ_{11}; All households: $\{h_u\}$; All schools: $\{sch_v\}$; All workplacs $\{w_z\}$; All neighborhoods: $\{nbhd_u\}$; ***Contact History List***.

Conditions. This function is active if and only if individual *i* is infectious and individual *j* is susceptible on the current day: $G_i(t)$=*Infectious* AND $G_i(\mathrm{t})$=*Susceptible*. If these conditions are

satisfied by current state of infectious individual $X_i(t)$ and current state of susceptible individual $X_j(t)$, then *Contact History List Update* is active and adds individuals *i* and *j* into the ***Contact History List*** as follows.

The structure of ***Contact History List***. ***Contact History List*** is a system level list in which given a date, a place, and an infectious case, all of its contacts can be retrieved. The structure of the list is shown in Figure 1. ***Contact History List*** has 5 levels of index. (1) Index level 1 is the **Day Index** in which there are 7 nodes (from 0 to 6) representing the current day, 1 day before the current day, …, 6 days before the current day. Each node links to its next level index of place type. (2) Index level 2 is the **Place Type Index** in which there are 6 nodes representing Household, School, Workplace, Classroom, Office and Neighborhood. Each of the place nodes links to its next level index of infectious place ID. (3) Index level 3 is the **Infectious Place ID Index** which has *m* nodes representing infectious places IDs. The "infectious place" means that at least one of individuals in this place is infectious. And each of *m* infectious places links to its next level index of infectious individuals in that place. (4) Index level 4 is **Infectors Index** which has many nodes indicating infectious individuals at the place indicated by **Infectious Place ID**, level 3 index. Each infector node links to its next level index of contacts. (5) The final level index is **Contact List Index** which saves all the contacts contacted by each the infectious individual linked from index level 4 (**Infectors Index**), at the place linked from index level 3 (**Infectious Place ID Index**), with the place type linked from level 2 (**Place Type Index**), on the day linked from index level 1 (**Day Index**).

A record in ***Contact History List*** can be written in this form:

$\boldsymbol{Contact_{record}}$ **= (Day Index, Place Type Index, Infectious Place ID Index, Infectors Index, Contact List Index).**

For example, $\boldsymbol{Contact_{record}} = (x_1, x_2, x_3, x_4, x_5)$ means that on day x_1, at place x_3 with place type x_2, infectious individual x_4 contacted individual x_5.

Details. To add infectious individual *i* and its contact *j*, we need to know (1) contact date, (2) the contact place ID, (3) the place type of contact place, (4) the infector ID and (5) the contact ID, and then add this information into the five-level indices. The contact date is the current day *t*, the place type is $\text{contact}_{\text{place}}(i, j)$, the infector is *i*, and the contact is *j*. The households set, the schools set, the daycares set, the workplaces set and the neighborhoods set can be used to find the IDs of contact places as stated in ***Initial Conditions***.

To update the ***Contact History List*** , the simulation programs first enter into the index level 1 the **Day Index** from the root node ***Contact History List***, and follow the *d* th (*d* = 7 modulo *t*) link to the **Place Type Index** (create a new Place Type Index if the link is empty). In the **Place Type Index**, the simulation programs find the node whose place type is $\text{contact}_{\text{place}}(i, j)$ (add a new node if type $\text{contact}_{\text{place}}(i, j)$ is not in the index level 2), and then follow its link to the **Infectious Place ID Index** (create a new infectious Place Index if the link is empty). In the **Infectious Place ID Index**, the simulation programs find the contact place ID and follow the link to the **Infectors Index** (if it is not in the index, then add the contact place ID, create a new Infectors Index and make it linked from the contact place ID node). In the **Infectors Index**: if it is the first time that individual *i* appears, the simulation programs add individual *i* into the index and create a new Contact List Index linked from individual *i*; otherwise, the simulation programs directly get the **Contact List Index** from existing individual *i*. In the **Contact List Index**, the simulation programs add individual *j* into the individual *i*'s **Contact List Index** if it is the first time *j* appears. In this way, a new record $\boldsymbol{Contact_{record}}=(t, \text{contact}_{\text{place}}(i, j), \text{contact place ID}, i, j)$ can be added into the ***Contact History List***.

In summary, *Contact History List Update* does not change states of infectious individual *i* and susceptible individual *j*. It updates the ***Contact History List*** by adding *i* and *j*, and makes them retrievable from their individuals' IDs, contact date and contact place.

4.5 Public Health Contact Tracing

Function. This function works on the population level. For each measles case in the population, it traces all contacts (from current day *t* to θ_{11} days ago) of the case, and then schedules the intervention date for each of the traced contacts.

Input parameters. Current day *t*; Current state for the population: $s(t) = (X_1(t), \ldots , X_j(t), \ldots X_N(t))$; Probability that an identified case recalls one of its contacts: θ_2 ; Delay (days) of intervention for a self-reported case: θ_3 ; Delay (days) of intervention for a traced case: θ_4; Delay (days) from start date of symptoms for a measles case to the date that the case visits doctor or hospital: θ_5; Probability that public health workers find out a contact of an identified case: θ_6; Days of tracing back: θ_{11}; Place of the contact tracing: θ_{12}; Public Health Contact Tracing indicator: θ_{20}; ***Contact History List***.

Conditions. This function is active if and only if the Public Health Contact Tracing indicator θ_{20}=TRUE.

Details. Given the state of population on current day $s(t)$, the *Public Health Contact Tracing* first reads each individual state $X_j(t)$, j=1, …, N. ; if individual *j* is infectious ($G_j(t)=I$), then the *Public Health Contact Tracing* needs to search the ***Contact History List*** to retrieve all contacts of individual *j* on the current day *t,* all contacts of individual *j* on day t-1 (if t-1>0), …, all contacts of individual *j* on the day $t - \theta_{11}$ (if $t - \theta_{11} >0$). The *Public Health Contact Tracing* also needs to schedule a date of ***Public Health Intervention*** for each of contacts of individual *j.*

Retrieving all contacts of individual j on the current day t and saving them into a list ***Contacts***$_j$(t) can be done as follows:

(1) In the **Day Index,** locate to d th (d = 7 modulo t) node, and get the **Place Type Index**.

(2) In the **Place Type Index,** for each place type in θ_{12} (initialized with 4 types: Household, School, Daycare, Workplace), get the **Infectious Place ID Index**.

(3) In the **Infectious Place ID Index**, get the **Infectors Index** for each infectious place.

(4) In the **Infector Index**, if individual j is in the list of infectors, then get individual j's **Contact List Index,** and add all contacts in the **Contact List Index** into the ***Contacts***$_j$(t).

(5) Repeat step (4) until each infectious place in step (3) has been searched. Now the ***Contacts***$_j$(t) has all contacts of individual j for only one place type.

(6) Repeat step (3) until each contact place type in step (2) has been searched. Now the ***Contacts***$_j$(t) already has all contacts of individual j, at Household, School, Daycare, and Workplace, on day t.

Repeating above steps from (1) to (6) with different tracing day t' (t' = t-1 (if t-1>0), …, $t - \theta_{11}$ (if $t - \theta_{11}$ >0)) can get: all contacts of individual j on current day t ***Contacts***$_j$(t), all contacts of individual j on the day t-1 ***Contacts***$_j$(t-1), …, and all contacts of individual j on the day $t - \theta_{11}$ ***Contacts***$_j$($t - \theta_{11}$).

On current day t, scheduling the ***Public Health Intervention*** for individual j, as well as for all contacts (in the ***Contacts***$_j$(t') on day t') of individual j, can be done as following steps:

(1) Schedule a new start date for the Public Health Intervention for the infectious case j. If individual j has never been scheduled for an intervention date ($V_i(t)$ = -1), a new intervention date may be $\theta_5 + \theta_3$ days after its start date of symptoms $Sym_i(t)$, because the self-report (that individual j reports itself to doctor or hospital due to the symptoms of measles) may occur on

several days (θ_5) after the first symptomatic date, and intervention for this self-reported case may start θ_3 days later from the reporting date ($Sym_i(t) + \theta_5$). Therefore, the new intervention date for individual j can be $Sym_i(t) + \theta_5 + \theta_3$. However, if current day t is not earlier than the assumed new intervention date ($t \geq Sym_i + \theta_5 + \theta_3$) which means j has not been given any intervention in $\theta_5 + \theta_3$ days after symptoms, then j should be given a proper intervention immediately and the new intervention day is today t. In the case that individual j's intervention date has been scheduled ($V_j(t) > -1$) previously, a replacement intervention date could be $Sym_j(t) + \theta_5 + \theta_3$ or today t (if current day is not earlier than the assumed intervention date $t \geq Sym_i + \theta_5 + \theta_3$). If the old scheduled intervention date $V_j(t)$ is not later than the replacement intervention date ($V_j(t) \leq Sym_i(t) + \theta_5 + \theta_3$ OR $V_j(t) \leq t$), then keep the old scheduled intervention date $V_j(t)$ unchanged because public health workers may give an intervention to individual j as earlier as possible to stop the measles. Before the intervention, public health workers may need to find individual j with probability θ_6. As a self-reported case, individual j's start date of ***Public Health Intervention*** can be updated by $V_j(t)$=

$$\begin{cases} Sym_j(t) + \theta_5 + \theta_3, \text{ if } (\, t < Sym_j(t) + \theta_5 + \theta_3 \,) \text{ AND } (\, Sym_j(t) + \theta_5 + \theta_3 < V_j(t) \text{ OR } V_j(t) = -1 \,) \text{ AND } \delta_{\mathrm{R}}(\theta_6) \\ t, \text{ if } (\, t \geq Sym_j(t) + \theta_5 + \theta_3 \,) \text{AND} (\, t < V_j(t) \text{ OR } V_j(t) = -1 \,) \text{AND} \delta_{\mathrm{R}}(\theta_6) \\ V_j(t), \text{ else} \end{cases}$$

(2) For each of contacts of individual j on day t in the ***Contacts****$_j$*(t), if the contact i has been exposed before current day t ($E_i(t) \leq t$), then an intervention for individual i should be scheduled. If individual i has never had a scheduled intervention date ($V_i(t) = -1$), a new intervention date may be scheduled θ_4 days after **individual *j***'s self-report date of symptoms $Sym_j(t) + \theta_5$, because only after the infectious case ***j*** reports itself to a doctor or a hospital, public health workers can start finding i out from the contact list of j, and intervention for this traced case may start θ_4 days later from primary case self-report date ($Sym_i(t) + \theta_5$). Therefore the new

intervention date for individual *i* can be $Sym_i(t) + \theta_5 + \theta_4$. However, if current day *t* is not earlier than the assumed new intervention date ($t \geq Sym_i + \theta_5 + \theta_4$) which means *i* has not been given any intervention in θ_4 days after the primary case's self-report date, then it should be given a proper intervention immediately, and the new intervention day is today *t.* In the case that individual *i*'s intervention date has been scheduled ($V_i(t) > -1$), a replacement intervention date could be $Sym_j(t) + \theta_5 + \theta_4$ or today *t* (if current day is not earlier than the assumed intervention date $t \geq Sym_i + \theta_5 + \theta_4$). If the old scheduled intervention date $V_i(t)$ is not later than the replacement intervention date ($V_i(t) \leq Sym_i(t) + \theta_5 + \theta_3$ OR $V_i(t) \leq t$), then keep the old scheduled intervention date $V_i(t)$ unchanged, so that public health workers can give the intervention to individual *i* as earlier as possible to prevent measles. Before the intervention, public health workers also need to find the primary case *j* with probability θ_6, and the primary case *j* may recall the contact *i* with probability θ_2, and public health workers again may find the contact *i* with probability θ_6. As a traced case, individual *i*'s start date for the ***Public Health Intervention*** can be scheduled by $V_i(t)$=

$$\begin{cases} Sym_j(t) + \theta_5 + \theta_3, \text{ if } (\, t < Sym_j(t) + \theta_5 + \theta_3 \,) \text{ AND } (\, Sym_j(t) + \theta_5 + \theta_3 < V_j(t) \text{ OR } V_j(t) = -1 \,) \text{ AND } \delta_R(\theta_6) \\ t, \text{ if } (\, t \geq Sym_j(t) + \theta_5 + \theta_3 \,) \text{AND} (\, t < V_j(t) \text{ OR } V_j(t) = -1 \,) \text{AND} \delta_R(\theta_6) \\ V_j(t), \text{ else} \end{cases}$$

Repeating above steps (1) and (2) with different contact day *t'* ($t' = t-1$ (if t-1>0), …, $t - \theta_{11}$ (if $t - \theta_{11} > 0$)) can schedule the ***Public Health Intervention*** start date for individual *j*, as well as for all contacts on current day *t*, on the day *t*-1 (if *t*-1>0), …, $t - \theta_{11}$ (if $t - \theta_{11} > 0$). An individual who already has the scheduled intervention date may be rescheduled with another intervention date, if the new intervention date is earlier than the old intervention date in order to schedule an intervention as early as possible.

In summary, the *Public Health Contact Tracing* repeats the above procedures for each individual j (j=1, …, N) to update j's intervention date to $\boldsymbol{V_j(t)}$, and to update each of j's contacts, i's intervention date to $\boldsymbol{V_i(t)}$. The population state on current day t will be updated by *Public Health Contact Tracing(* $s(t)$) = *Public Health Contact Tracing* ($X_1(t)$, … , $X_j(t)$, … $X_N(t)$) = ($\boldsymbol{V_1(t)}$, **…** , $\boldsymbol{V_j(t)}$, **…** $\boldsymbol{V_N(t)}$).

4.6 Public Health Intervention

Function. This is an individual-level function. Based on input from the individual's current state, it gives the individual an intervention (PEP MMR Vaccine, PEP IG, and Home Quarantine).

Input parameters. Current day t; Current state for individual j, $X_j(t)$ = ($G_j(t)$, $E_j(t)$, $I_j(t)$, $Sym_j(t)$, $R_j(t)$, $P_j(t)$, $A_j(t)$, $V_j(t)$, H_j(t), $Sch_j(t)$, $Daycare_j(t)$, $W_j(t)$, θ); Intervention strategies: θ_{13} ; Public Health Intervention indicator: θ_{21} ; Probability that an individual is cooperative with public health workers who are providing interventions: θ_7 ; Probability that an individual complies with the intervention of Home Quarantine: θ_1 ; Efficacy of PEP MMR Vaccine (one dose) θ_8; Efficacy of PEP IG: θ_9.

Conditions. This function is active if and only if: the Public Health indicator is active (θ_{21}=TRUE), AND, there is at least one intervention in the intervention strategies (θ_{13}=000), AND, before intervention individual j is in the course of infection ($E_j(t) \leq t < R_j(t)$). If all of these conditions are satisfied, the Public Health Intervention will update the state of individual j as follows.

Details. One intervention (PEP MMR Vaccine, PEP IG or Home Quarantine) will be selected based on the current day's state of individual *j*. If symptoms have not occurred on the current day ($E_j(t) \leq t < Sym_j(t)$), and the current day is not more than 3 days after the start date of exposure ($t - E_j(t) \leq 3$ days), and individual *j* is more than one year old ($A_j(t) > 1$ year), then one dose of PEP MMR vaccine will be given to individual *j*. The PEP IG will be given under either of these two circumstances: (1) symptoms have not occurred on current day ($E_j(t) \leq t < Sym_j(t)$), and current day *t* is more than 3 days but not more than 6 days after the start date of exposure (3 days < $t - E_j(t) \leq 6$ days); (2) symptoms have not occurred on current day ($E_j(t) \leq t < Sym_j(t)$), and the current day is not more than 6 days after the start date of exposure ($t - E_j(t) \leq 6$ days), and individual *j*'s age is not more than 1 year ($A_j(t) \leq 1$ year). If symptoms occurred before the current day ($Sym_j(t) \leq t$), then Home Quarantine will be chosen. The intervention selection can be summarized as a function: $select_{intervention}(X_j(t))$=

$$\begin{cases} \text{Home Quarantine}, & Sym_j(t) \leq t \\ \text{PEP MMR Vaccnine}, & t \leq Sym_j(t) \text{ AND } t - E_j(t) \leq 3 \text{ AND } A_j(t) > 1 \\ \text{PEP IG}, & (t \leq Sym_j(t) \text{ AND } 3 < t - E_j(t) \leq 6) \text{ OR } (t \leq Sym_j(t) \text{ AND } t - E_j(t) \leq 6 \text{ AND } A_j(t) \leq 1) \end{cases} .$$

After intervention method has been selected, individual *j* may accept the intervention with probability θ_7 (for PEP MMR Vaccine and PEP IG) or θ_1 (for Home Quarantine):

$prob_{accept}(X_j(t)) = \begin{cases} \theta_7, select_{intervention}(X_j) = \text{PEP MMRVaccine or PEP IG} \\ \theta_1, select_{intervention}(X_j) = \text{Home Quarantine} \end{cases}$; and a random number R between 0 and 1 is generated to decide whether to accept the intervention:

$\delta_R(prob_{accept}(X_j(t))) = \begin{cases} 1, R \leq prob_{accept}(X_j(t)) \\ 0, R > prob_{accept}(X_j(t)) \end{cases}$. If individual *j* accepts PEP MMR Vaccine ($select_{intervention}(X_j(t))$=PEP MMR Vaccine AND $\delta_R(prob_{accept}(X_j(t)))=1$), and if this intervention is effective ($\delta_R(\theta_8)=1$), then the stage of measles will be updated to recovery $G_j(t)=R$, recovery

date will be adjusted to current day $R_j(t)=t$, and the immunity protection should be increased to the level of effectiveness of one dose MMR Vaccine $P_j(t)=\theta_8$. If individual *j* accepts PEP IG ($select_{intervention}(X_j(t))$=PEP IG AND $\delta_R(prob_{accept}(X_j(t)))=1$), and if this intervention is effective ($\delta_R(\theta_9)=1$), then the stage of measles will be updated to recovery $G_j(t)=R$ and the recovery date will be adjusted to the current day $R_j(t)=t$, with the immunity protection increased to the level of efficacy of PEP MMR Vaccine $P_j(t)=\theta_9$.

If Home Quarantine is the selected intervention and individual *j* is willing to stay at home ($select_{intervention}(X_j(t))$=Home Quarantine AND $\delta_R(prob_{accept}(X_j(t)))=1$), then the stage of measles $G_j(t)$ and recovery date $G_j(t)$ are **unchanged**, but individual *j* will be tagged with ***stay home*** until the recovery date.

Another case when Home Quarantine will be selected is when PEP MMR Vaccine or PEP Vaccine has been selected but individual *j* refuses to accept the intervention, so the only choice the public health workers have is to recommend Home Quarantine for individual *j*. The condition for this case can be expressed by ($select_{intervention}(X_j(t))$=PEP MMR Vaccine AND $\delta_R(prob_{accept}(X_j(t)))=0$) OR ($select_{intervention}(X_j(t))$=PEP IG AND $\delta_R(prob_{accept}(X_j(t)))=0$). In this case, if individual *j* is willing to stay home with the probability Home Quarantine θ_1, it will be tagged with ***stay home*** until its recovery date.

In summary, *Public Health Intervention* changes individual *j*'s state of measles stage and immunity protection if PEP MMR Vaccine or PEP IG is selected as the intervention method. If Home Quarantine is selected, *Public Health Intervention* tags ***stay home*** on individual *j* until its recovery date.

$G_j(t) = R$, if ($select_{intervention}(X_j(t))$=PEP MMR Vaccine AND $\delta_R(prob_{accept}(X_j(t)))=1$ AND $\delta_R(\theta_8)=1$) OR ($select_{intervention}(X_j(t))$=PEP IG AND $\delta_R(prob_{accept}(X_j(t)))=1$ AND $\delta_R(\theta_9)=1$);

$G_j(t) = G_j(t)$, else.

$P_j(t) = \theta_8$, if ($select_{intervention}(X_j(t))$=PEP MMR Vaccine AND $\delta_R(prob_{accept}(X_j(t)))=1$ AND $\delta_R(\theta_8)=1$);

$P_j(t) = \theta_9$, if ($select_{intervention}(X_j(t))$=PEP IG AND $\delta_R(prob_{accept}(X_j(t)))=1$ AND $\delta_R(\theta_9)=1$);

$P_j(t) = P_j(t)$, else.

stay home = **TRUE**, if ($select_{intervention}(X_j(t))$=Home Quarantine AND $\delta_R(prob_{accept}(X_j(t)))=1$) OR ($select_{intervention}(X_j(t))$=PEP MMR Vaccine AND $\delta_R(prob_{accept}(X_j(t)))=0$ AND $\delta_R(\theta_1)$) OR ($select_{intervention}(X_j(t))$=PEP IG AND $\delta_R(prob_{accept}(X_j(t)))=0$ AND $\delta_R(\theta_1)$);

stay home = FALSE, else.

Therefore, individual *j*'s state for current day *t* is updated by *Public Health Intervention* ($X_j(t)$) = ($\boldsymbol{G_j(t)}$, $\boldsymbol{P_j(t)}$). More detailed algorithm flow chart for *Public Health Intervention* on an individual is shown in Figure 2. The ***stay home*** tag will be used to choose contacts of individual *j* in ***System Update***.

4.7 Next Day

Function. This function works on the population level. It happens per day when all events for individuals and the population on a day have been completed. It updates each individual's current day's state to the next day's state, and updates current day *t* to next day $t=t+1$.

Input parameters. Current day *t*; Current state for the population: $s(t) = (X_1(t), \ldots , X_j(t), \ldots X_N(t))$; The end day of simulation: θ_{24}.

Conditions. This function is active if and only if $t \le \theta_{24}$.

Details. Given the population state on current day *t, s*(*t*), the *Next Day* updates each individual's state by $X_j(t+1) = X_j(t)$, and updates the current day *t* to the next day $t = t+1$. The population state will be updated by:

Next Day($s(t)$) = *Next Day*($X_1(t)$, … , $X_j(t)$, … $X_N(t)$) = $s(t+1)$ = ($X_1(t+1)$, … , $X_j(t+1)$, … $X_N(t+1)$)=

$$\begin{pmatrix} G_1(t+1) & E_1(t+1) & I_1(t+1) & Sym_1(t+1) & R_1(t+1) & P_1(t+1) & V_1(t+1) & A_1(t+1) & H_1(t+1) & Sch_1(t+1) & W_1(t+1) \\ \vdots & & & & & & & & & & \vdots \\ G_N(t+1) & E_N(t+1) & I_N(t+1) & Sym_N(t+1) & R_N(t+1) & P_N(t+1) & V_N(t+1) & A_N(t+1) & H_N(t+1) & Sch_N(t+1) & W_N(t+1) \end{pmatrix}$$

4.8 System Update

Function. This function works on the population level, given the initial population state $s(t=0)$. It updates the population state from the first day t=0 to the last day t= θ_{24} by running the above events/functions and applying them to each individual.

Input parameters. Current day t; Current state for the population: $s(t)$ = ($X_1(t)$, … , $X_j(t)$, … $X_N(t)$); The end day of simulation: θ_{24}. All households: $\{h_u\}$; All schools: $\{sch_v\}$; All workplacs $\{w_z\}$; All neighborhoods: $\{nbhd_u\}$; ***Contact History List*** (empty on day 0).

Conditions. This function is active if and only if $t \leq \theta_{24}$.

Details. Given the population with initial state on day 0, $s(t=0)$, the *System Update* will dynamically change the population state on each day by following steps.

(1) **BEGIN Update** of the population health state $s(t)$ on day t.

(2) For each state of individual in the population, $X_j(t)$, do ***Health Update($X_j(t)$)***

(3) In the ***Health Update***, if it is time to give individual j an intervention ($t= V_j(t)$), then do ***Public Health Intervention***($X_j(t)$) to update his/her immunity $\boldsymbol{P_j(t)}$ and measles stage $\boldsymbol{G_j(t)}$. The measles stage $\boldsymbol{G_j(t)}$ will also be updated by ***Health Update***($X_j(t)$).

(4) **END Update** population health state for day t.

(5) **BEGIN Measles transmission** in the population for day t.

(6) For each Household $h_u \in \{h_u\}$, u = 1, 2, …, $N_{household}$, do steps (7), (8) and (9). **(Household Transmission)**

(7) For each infectious individual j in h_u, the random household contacts for individual j are generated as follows:

> (7.1) For each j's household members in h_u, if the selected individual is susceptible, then the individual will become j's contact by $\delta_R(\theta_{14})$, where θ_{14} is the daily contact probability between any two household members.
>
> (7.2) Add all random household contacts of individual j into $Contacts_{household}(j)$.

(8) For each contact i of j in $Contacts_{household}(j)$, do ***Transmission*** **($X_j(t)$, $X_i(t)$)**, in which the infectious j contacts the susceptible household member i.

(9) In ***Transmission*** **($X_j(t)$, $X_i(t)$)**: do ***Contact History List Update*** **($X_j(t)$, $X_i(t)$)** to save the contact information in the ***Contact History List***; if it is a **successful transmission**, update the new infected individual i's exposed start date **$E_i(t)$** and measles stage **$G_i(t)$,** and then do ***Trajectory*****($X_i(t)$)** to predict and update i's infectious start date **$I_i(t)$**, and recovery start date **$R_i(t)$**.

(10) For each School $sch_v \in \{sch_v\}$, v = 1, 2, …, N_{school}, do steps (11), (12) and (13). **(School Transmission)**

(11) For each infectious individual j in sch_v, if j is not labeled with ***stay home***, then the random school contacts for individual j are generated as follows:

(11.1) Randomly pick up θ_{15} individuals in sch_v, where θ_{15} is school contact rates. For each of θ_{15} selected individuals, if the selected individual is susceptible, then the individual will become *j*'s contact.

(11.2) Add all random susceptible school contacts of individual *j* into $Contacts_{school}(j)$.

(12) For each contact *i* of *j* in $Contacts_{school}(j)$, do ***Transmission*** **($X_j(t)$, $X_i(t)$)**, where the infectious *j* contacts the susceptible schoolmate *i*.

(13) In ***Transmission*** **($X_j(t)$, $X_i(t)$)**: do ***Contact History List Update*** **($X_j(t)$, $X_i(t)$)** to save the contact information in the ***Contact History List***; if it is a **successful transmission**, update the new infected individual *i*'s exposed start date **$E_i(t)$** and measles stage **$G_i(t)$,** and then do ***Trajectory*****($X_i(t)$)** to predict and update *i*'s infectious start date **$I_i(t)$**, and recovery start date **$R_i(t)$**.

(14) For each Workplace $w_z \in \{w_z\}$, $z = 1, 2, \ldots, N_{workplace}$, do steps (15), (16) and (17).

(Workplace Transmission)

(15) For each infectious individual *j* in W_z, if *j* is not labeled with ***stay home***, then the random workplace contacts for individual *j* are generated as follows:

(15.1) Randomly pick up θ_{16} individuals in W_z, where θ_{16} is workplace contact rates. For each of θ_{16} selected individuals, if the selected individual is susceptible, then the individual will become one of *j*'s contacts.

(15.2) Add all random susceptible workplace contacts of individual *j* into $Contacts_{workplace}(j)$.

(16) For each contact *i* of *j* in $Contacts_{workplace}(j)$, do ***Transmission*** **($X_j(t)$, $X_i(t)$)**, where the infectious *j* contacts the susceptible workmate *i*.

(17) In ***Transmission*** **($X_j(t)$, $X_i(t)$)**: do ***Contact History List Update*** **($X_j(t)$, $X_i(t)$)** to save this contact information into the ***Contact History List***; if it is a **successful transmission**, update the new infected individual *i*'s exposed start date **$E_i(t)$** and measles stage **$G_i(t)$,** and then do ***Trajectory*****($X_i(t)$)** to predict and update *i*'s infectious start date **$I_i(t)$** and recovery start date **$R_i(t)$**.

(18) For each Neighborhood $nbhd_z \in \{nbhd_z\}$, $z = 1, 2, \ldots, N_{neighborhood}$, do steps (19), (20) and (21). **(Neighborhood Transmission)**

(19) For each infectious individual *j* in $nbhd_z$, if *j* is not labeled with ***stay home**,* then the random neighborhood contacts for individual *j* are generated as follows:

(19.1) A random neighborhood for individual *j* can be the home neighborhood, one of the surrounding neighborhoods, or one of the community neighborhoods. Home neighborhood is where individual *j*'s household is located. The surrounding neighborhoods are eight adjacent neighborhoods around j's home neighborhood. The community neighborhoods are all neighborhoods within radius of θ_{18} km from *j*'s household. A random neighborhood $nbhd_r$ for individual *j* is picked with the highest probability that *j* visits the home neighborhood, and a lesser probability that *j* visits one of the surrounding neighborhoods, and a small probability of visiting one of the community neighborhoods.

(19.2) Randomly pick up θ_{17} individuals in $nbhd_r$, where θ_{17} is neighborhood contact rates. For each of θ_{17} selected individuals, if the selected individual is susceptible, then the individual will become one of *j*'s contacts.

(19.3) Add all random susceptible neighborhood contacts of individual *j* into $Contacts_{neighborhood}(j)$.

(20) For each contact *i* of *j* in $Contacts_{neighborhood}(j)$, do ***Transmission*** **($X_j(t)$, $X_i(t)$)**, where the infectious *j* contacts the susceptible neighborhood contact *i*.

(21) In ***Transmission*** **($X_j(t)$, $X_i(t)$)**: do ***Contact History List Update*** **($X_j(t)$, $X_i(t)$)** to save this contact information in the ***Contact History List***; if it is a **successful transmission**, update the new infected individual *i*'s exposed start date $E_i(t)$ and measles stage $G_i(t)$, and then do ***Trajectory***(**$X_i(t)$**) to predict and update *i*'s infectious start date $I_i(t)$ and recovery start date $R_i(t)$.

(22) **END Measles transmission** in the population for day *t*.

(23) **BEGIN Contact tracing** in the population for day *t*.

(24) Do ***Public Health Contact Tracing*** **($X_1(t)$, … , $X_j(t)$, … $X_N(t)$)** to find all contacts (on the current day and in past θ_{11} days) of each infectious individual, and update their start dates of ***Public Health Intervention***, ($V_1(t)$, … , $V_j(t)$, … $V_N(t)$).

(25) **END Contact tracing** in population for day *t*.

(27) **BEGIN Update** the population state from day t to *t*+1.

(28) Do ***Next Day***(**$X_1(t)$, … , $X_j(t)$, … $X_N(t)$**). For each individual *j* (all active events that may change individual *j*'s state have been updated for the current day *t by above steps*) assign *j*'s updated state as *j*'s start state for the next day: $X_j(t+1)= X_j(t)$. And update simulation day to next day: $t = t+1$.

(29) **END Update** the population state from day *t* to *t*+1.

(30) IF $t \leqslant \theta_{24}$ Go to step (1) with updated *s*(*t*) and *t*.

(31) **END SIMULATION** if t > θ_{24}.

Table 1: State of each individual in the model.

Variable	Interpretations
G_j	Stage of measles for individual *j*: Susceptible, Exposed, Infectious, and Recovered.
E_j	Start date of exposure for individual *j*.
I_j	Start date of infectiousness for individual *j*.
Sym_j	Start date of symptoms for individual *j*.
R_j	Start date of recovery for individual *j*.
P_j	Protection by individual *j*'s immunity because of previous vaccinations.
V_j	Start date of Public Health intervention for individual *j*.
A_j	Age of individual *j*.
H_j	Household location for individual *j*; it is a vector with 3 elements: the unique ID, longitude, and latitude.
Sch_j	School location for individual *j*; it is a vector with 3 elements: the unique ID, longitude, and latitude.
$Daycare_j$	Daycare location for individual *j*; it is a vector with 3 elements: the unique ID, longitude, and latitude.
W_j	Workplace location for individual *j*; it is a vector with 3 elements: the unique ID, longitude, and latitude.

Table 2: Global variables in the model.

Variable	Interpretations	Values			References
		Initial	Lower	Upper	
θ_1	Probability that an individual complies with the intervention of Home Quarantine.	0.97	0.9	1	Assume
θ_2	Probability that an identified case recalls one of its contacts.	1	1	1	Assume
θ_3	Delay (days) of intervention for a self-reported case.	1	1	3	Assume
θ_4	Delay (days) of intervention for a traced case.	1	1	3	Assume
θ_5	Delay (days) from start date of symptoms for a measles case to the date that the case visits doctor or hospital.	2	1	6	Assume
θ_6	Probability that public health workers find out a contact of an identified case.	1	0.7	1	Assume
θ_7	Probability that a person investigated is cooperative with public health workers who are providing interventions.	1	1	1	Assume
θ_8	Efficacy of Post Exposure Prophylaxis (PEP) MMR Vaccine.	0.93	0.92	0.95	[9, 10]
θ_9	Efficacy of PEP IG.	0.75	0.6	0.9	[11-13]
θ_{10}	Efficacy of mass Vaccine (two-dose).	0.99	0.99	0.99	[9, 10]
θ_{11}	Days of tracing back (excluding current day)	6	6	6	Assume
θ_{12}	Place of the contact tracing; it indicates the places in which contacts of an identified case are possible to be traced. An indicator with 3 binary codes: from 0000 to 1111. “0000” means no place is traceable, and “1111” means contacts in household, school, daycare and workplace are traceable.	1111	0000	1111	Assume
θ_{13}	Intervention strategies: 8 combinations of PEP Vaccine, PEP IG, and Home quarantine.	111	000	111	Assume

	Values (3 binary codes) are from 000 to 111 corresponding to applying no intervention and apply all three interventions.				
θ_{14}	Probability that household members contact each other every day.	0.46	0.01	1	Assume
θ_{15}	Contact rates per day in School.	9	3	20	Assume
θ_{16}	Contact rates per day in Workplace.	3	0	7	Assume
θ_{17}	Contact rates per day in Neighborhood.	3	0.5	7	Assume
θ_{18}	Radius (kilometers) of community from household. During a given day, individual *j* may visit one of neighborhoods within θ_{18} km from the household of individual *j*.	20	5	50	Assume
θ_{19}	Previous Mass vaccine indicator (1 binary code), which indicates whether or not the population were initialized with previous vaccine coverage.	1	0	1	Assume
θ_{20}	Public Health Contact Tracing indicator (1 binary code), which indicates whether or not the model enables the contact tracing.	1	0	1	Assume
θ_{21}	Public Health Intervention indicator (1 binary code), which indicates whether not the model enables the interventions.	1	0	1	Assume
θ_{22}	Probability of Infectivity during the course of infectiousness of a measles case.	1	0	1	Assume
θ_{23}	Number of index cases on initial day *t*=0.	1	1	1	Assume
θ_{24}	The end day of simulation.	365	1	600	Assume
θ_{25}	Transmission probability per contact in Household.	0.99	0.99	0.99	Assume
θ_{26}	Transmission probability per contact in School.	0.99	0.99	0.99	Assume

θ_{27}	Transmission probability per contact in Workplace.	0.99	0.99	0.99	Assume
θ_{28}	Transmission probability per contact in Neighborhood.	0.99	0.99	0.99	Assume
θ_{29}	The daily probability that an infectious case is willing to stay at home from the start date of his/her symptoms to the date of his/her recovery	0.61	0	1	Assume
θ_{30}	Contact rates per day in Daycares.	9	3	20	Assume
θ_{31}	Transmission probability per contact in Daycares.	0.99	0.99	0.99	Assume
θ_{32}	County ID: indicator of the county in current simulation.	1	1	58	There are 58 counties in California.

Table 3: The distributions of incubation, latent, infectious and symptomatic periods, and age-specific vaccination coverage used in the model.

Distribution	Interpretations	Values	References	Notes
π_1	Incubation period distribution (cdf): x_i is the probability that the *i* th day is the last day of incubation; and a measles case will become symptomatic within *n* days with 100% chance.	$\pi_1 = (x_0, x_1, \cdots, x_n)$. $0 \le x_i \le 1, 0 \le i \le n, \ x_n = 1.$ π1 = *(0, 0, 0, 0, 0, 0.1, 0.2, 0.6, 1)*	[14]	The start date of SYMPTOMS for each individual is generated by this distribution.
π_2	Latent period distribution (cdf): x_i is the probability that the *i* th day is the last day of latent; and a measles case will become infectious within *n* days.	$\pi_2 = (x_0, x_1, \cdots, x_n)$. $0 \le x_i \le 1, 0 \le i \le n, \ x_n = 1.$ Π2 = *(0, 0, 0, 0, 0, 0.1, 0.2, 0.6, 1)*	Assume	The start date of INFECTIOUSNESS for each individual is generated by this distribution.
π_3	Infectious period distribution (cdf): x_i is the probability that the *i* th day is the last day of infectiousness; and a measles case will become recovered within *n* days.	$\pi_3 = (x_0, x_1, \cdots, x_n)$. $0 \le x_i \le 1, \ 0 \le i \le n, x_n = 1$ Π3 = *(0, 0 ,0, 0, 0, 0, 0.3, 0. 7, 1)*	Assume	The start date of RECOVERY for each individual is generated by this distribution.
π_4	Symptomatic period distribution (cdf): x_i is the probability that the *i* th day is the last day of infectiousness; and a measles case will become recovered within *n* days.	$\pi_4 = (x_0, x_1, \cdots, x_n)$. $0 \le x_i \le 1, \ 0 \le i \le n, x_n = 1$ π4 = *(0, 0 ,0, 0, 0, 0, 0.3, 0. 7, 1)*	[14]	The start date of RECOVERY for each individual is generated by this distribution.

π_5	Distribution of vaccine coverage for every age (0 to 100): x_i is the coverage rate that people at age *i* are covered by mass two-dose MMR vaccination.	$\pi_5 = (x_0, x_1, \cdots, x_n)$. $0 \le x_i \le 1, 0 \le i \le n$.	Assume for age <= 18 years	At the beginning of simulation, the previous IMMUNITY for each individual is generated by the distribution given in Table 5 for individuals greater than 18 years of age.

Table 4: Active events/functions.

**Event/Function**	**Interpretations**	**Level**	**Input Variables**	**Conditions under which event/function is active**
Health Update	To update measles stage for individual *j*: Susceptible, Exposed, Infectious, and Recovered.	Individual	*individual j,* *current day t.*	$t = E_j$ *or* $t=I_j$ *or* $t=R_j$ *or* $t=V_j$.
Transmission	To try to transmit measles from an infectious individual *i* to a susceptible individual *j*.	Individual	*Infectious individual i,* *susceptible individual j,* *contact place*, *current time t,* $\theta_{22}, \theta_{25}, \theta_{26}, \theta_{27}, \theta_{28}, \theta_{31}$.	G_i=Infectious and $I_i \leq t < R_i$ and G_j=Susceptible and $I_j \leq t < R_j$.
Trajectory	To schedule start dates of infectiousness, symptoms and recovery for new infected individual *j*.	Individual	*individual j,* *current day t,* *Incubation period,* *Latent period,* *Infectious period.*	G_j=*Exposed* and $t = E_j$.
Contact History List Update	To record where and when an infectious individual *i* contacts a susceptible individual *j*.	Individual	*Infectious individual i,* *Susceptible individual j,* *current day t,* *contact place,* *Contact History List.*	G_i=Infectious and $I_i \leq t < R_i$.
Public Health Contact Tracing	To trace all contacts (from current day *t* to θ_{11} days ago) of each measles case, then schedule intervention date for each traced contacts.	Population	*population state s,* *current time t,* *Contact History List,* $\theta_2, \theta_3, \theta_4, \theta_5, \theta_{11}, \theta_{12}, \theta_{20}, \theta_{30}$.	*Contact Tracing indicator* θ_{20} =TRUE.
Public Health Intervention	To give individual *j* an intervention of PEP MMR Vaccine, PEP IG, and Home Quarantine.	Individual	*individual j,* *current day t,* $\theta_1, \theta_7, \theta_8, \theta_9, \theta_{13}, \theta_{21}$.	$E_j \leq t < R_j$ and $t=V_j$ and θ_{20} =TRUE and θ_{13} !=000.
Next Day	To update current day to next day, *t*=*t*+1.	-	*current day t*	Always active ($t \leq \theta_{24}$).
System Update	To update population state *s* on day *t* by organizing above	Population	*population state s,* *current day t,* *end day* θ_{24}.	Always active ($t \leq \theta_{24}$).

	events/functions and applying them to each individual.			

Table 5. Age-Specific Seroprevalence of Measles Antibody

Birth Cohort	Age (Years)	Measles Antibody Seroprevalence Estimate	Reference
1993	19	97.70%	McQuillan JID 2007[15] , CDC 2012 Elimination Report[16]
1992	20	97.70%	McQuillan JID 2007[15] , CDC 2012 Elimination Report[16]
1991	21	97.70%	McQuillan JID 2007[15] , CDC 2012 Elimination Report[16]
1990	22	97.70%	McQuillan JID 2007[15] , CDC 2012 Elimination Report[16]
1989	23	97.70%	McQuillan JID 2007[15] , CDC 2012 Elimination Report[16]
1988	24	97.70%	McQuillan JID 2007[15] , CDC 2012 Elimination Report[16]
1987	25	97.70%	McQuillan JID 2007[15] , CDC 2012 Elimination Report[16]
1986	26	96.40%	McQuillan JID 2007[15] , CDC 2012 Elimination Report[16]
1985	27	96.40%	McQuillan JID 2007[15] , CDC 2012 Elimination Report[16]
1984	28	96.40%	McQuillan JID 2007[15] , CDC 2012 Elimination Report[16]
1983	29	96.40%	McQuillan JID 2007[15] , CDC 2012 Elimination Report[16]
1982	30	96.40%	McQuillan JID 2007[15] , CDC 2012 Elimination Report[16]
1981	31	96.40%	McQuillan JID 2007[15] , CDC 2012 Elimination Report[16]
1980	32	96.40%	McQuillan JID 2007[15] , CDC 2012 Elimination Report[16]
1979	33	96.40%	McQuillan JID 2007[15] , CDC 2012 Elimination Report[16]
1978	34	96.40%	McQuillan JID 2007[15] , CDC 2012 Elimination Report[16]
1977	35	96.40%	McQuillan JID 2007[15] , CDC 2012 Elimination Report[16]
1976	36	92.40%	McQuillan JID 2007[15] , CDC 2012 Elimination Report[16]
1975	37	92.40%	McQuillan JID 2007[15] , CDC 2012 Elimination Report[16]
1974	38	92.40%	McQuillan JID 2007[15] , CDC 2012 Elimination Report[16]
1973	39	92.40%	McQuillan JID 2007[15] , CDC 2012 Elimination Report[16]
1972	40	92.40%	McQuillan JID 2007[15] , CDC 2012 Elimination Report[16]
1971	41	92.40%	McQuillan JID 2007[15] , CDC 2012 Elimination Report[16]
1970	42	92.40%	McQuillan JID 2007[15] , CDC 2012 Elimination Report[16]
1969	43	92.40%	McQuillan JID 2007[15] , CDC 2012 Elimination Report[16]
1968	44	92.40%	McQuillan JID 2007[15] , CDC 2012 Elimination Report[16]
1967	45	92.40%	McQuillan JID 2007[15] , CDC 2012 Elimination Report[16]
1966	46	96.60%	McQuillan JID 2007[15] , CDC 2012 Elimination Report[16]
1965	47	96.60%	McQuillan JID 2007[15] , CDC 2012 Elimination Report[16]
1964	48	96.60%	McQuillan JID 2007[15] , CDC 2012 Elimination Report[16]
1963	49	96.60%	McQuillan JID 2007[15] , CDC 2012 Elimination Report[16]
1962	50	96.60%	McQuillan JID 2007[15] , CDC 2012 Elimination Report[16]
1961	51	96.60%	McQuillan JID 2007[15] , CDC 2012 Elimination Report[16]

1960	52	96.60%	McQuillan JID 2007[15] , CDC 2012 Elimination Report[16]
1959	53	96.60%	McQuillan JID 2007[15] , CDC 2012 Elimination Report[16]
1958	54	96.60%	McQuillan JID 2007[15] , CDC 2012 Elimination Report[16]
1957	55	96.60%	McQuillan JID 2007[15] , CDC 2012 Elimination Report[16]
1956	56	96.60%	McQuillan JID 2007[15] , CDC 2012 Elimination Report[16]
1955	57	96.60%	McQuillan JID 2007[15] , CDC 2012 Elimination Report[16]
1954	58	96.60%	McQuillan JID 2007[15] , CDC 2012 Elimination Report[16]
1953	59	96.60%	McQuillan JID 2007[15] , CDC 2012 Elimination Report[16]
1952	60	96.60%	McQuillan JID 2007[15] , CDC 2012 Elimination Report[16]
1951	61	96.60%	McQuillan JID 2007[15] , CDC 2012 Elimination Report[16]
1950	62	96.60%	McQuillan JID 2007[15] , CDC 2012 Elimination Report[16]
1949	63	96.60%	McQuillan JID 2007[15], CDC 2012 Elimination Report[16]

Figure 1: Contact History List

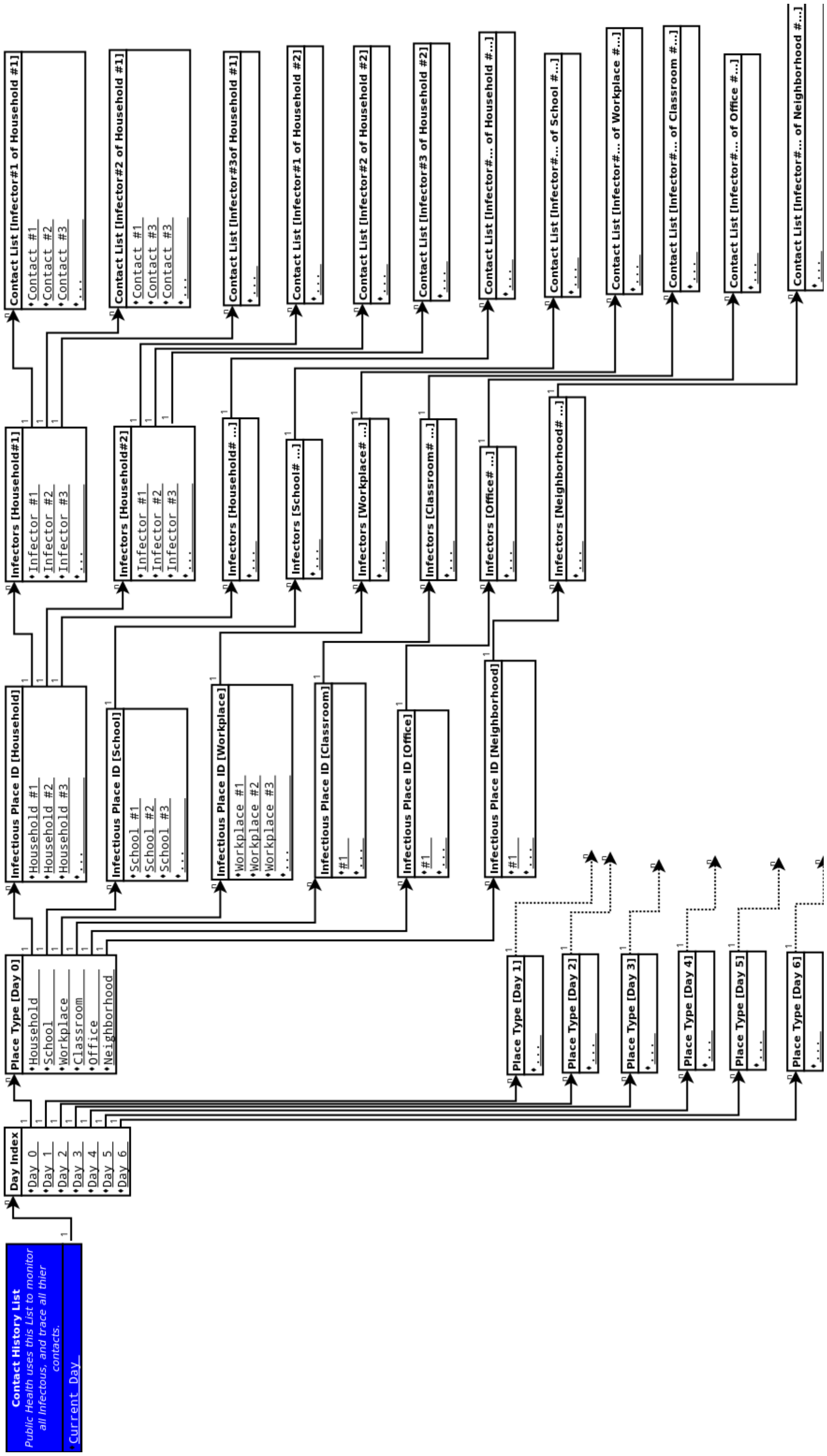

Figure 2: Public Health Intervention – algorithm flowchart.

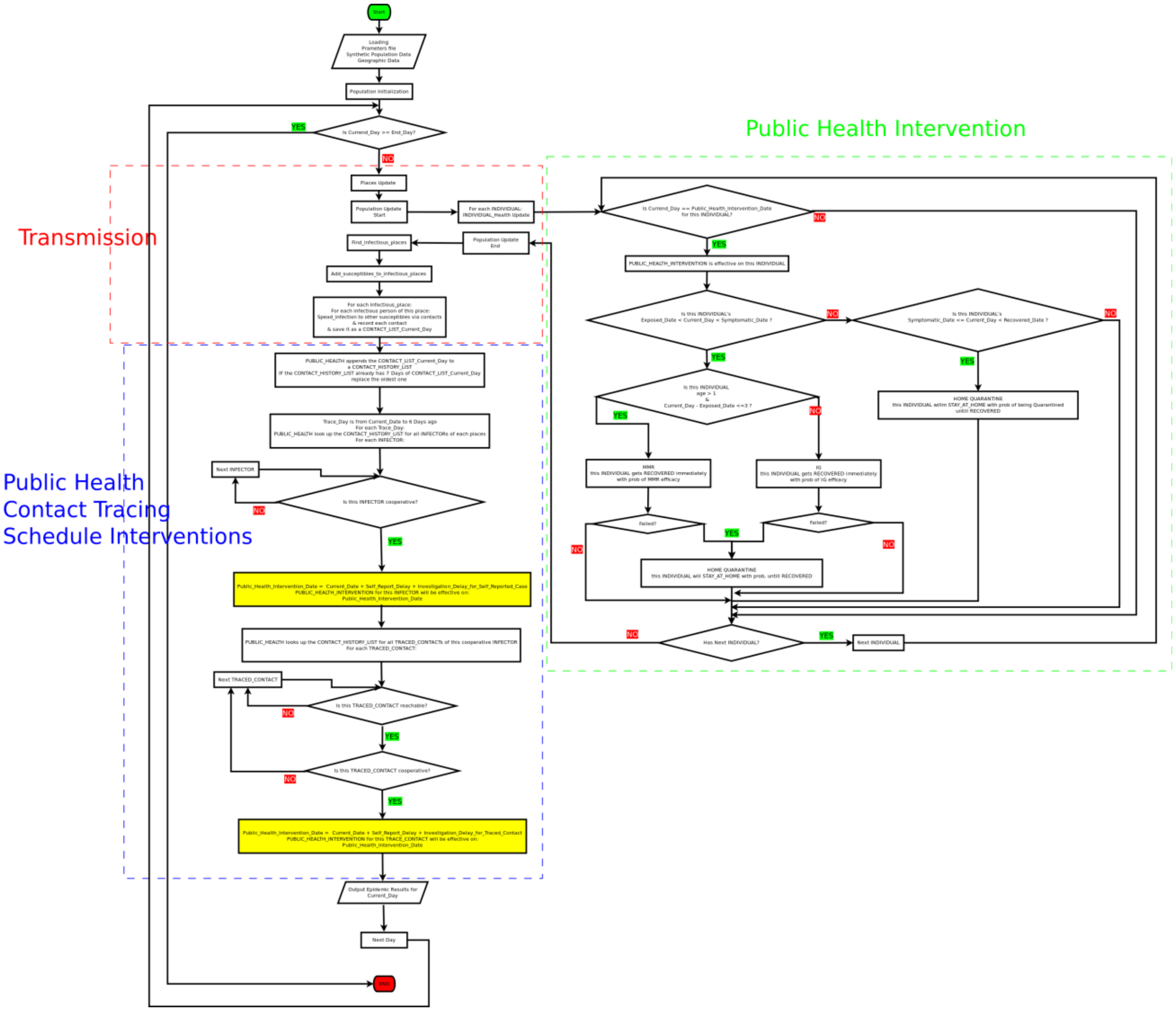

References

1. Macal CM, North MJ. Tutorial on agent-based modelling and simulation. Journal of Simulation. 2010;4(3):151-62.
2. Jennings NR. Agent-oriented software engineering. Lect Notes Artif Int. 1999;1611:4-10. PubMed PMID: ISI:000086482000002.
3. Jennings NR. On agent-based software engineering. Artif Intell. 2000;117(2):277-96. PubMed PMID: ISI:000085953000004.
4. Glasserman P, Yao DD. Monotonicity in generalized semi-Markov processes. Mathematics of Operations Research. 1992:1-21.
5. Health CDoP. Measles Investigation Quicksheet. 2013.
6. Grefenstette J, Brown S, DePasse J, Galloway D, Lee B, Weng Y-T, et al. FRED User's Guide. Version 27 January 2012. University of Pittsburgh and Carnegie-Mellon University and Pittsburgh Supercomputing Center and RTI International, 2012.
7. Grefenstette JJ, Brown ST, Rosenfeld R, Depasse J, Stone NT, Cooley PC, et al. FRED (A Framework for Reconstructing Epidemic Dynamics): an open-source software system for modeling infectious diseases and control strategies using census-based populations. BMC Public Health. 2013;13:940.
8. Wheaton W. U.S. Synthetic Population Database 2005-2009: Quick Start Guide. RTI International. 2012.
9. Uzicanin A, Zimmerman L. Field effectiveness of live attenuated measles-containing vaccines: a review of published literature. J Infect Dis. 2011;204 Suppl 1:S133-S48.
10. Demicheli V, Jefferson T, Rivetti A, Price D. Vaccines for measles, mumps and rubella in children. Cochrane Database Syst Rev. 2005;(4):CD004407.
11. Ordman CW, Jennings CG, Janeway CA. Chemical, clinical, and immunological studies on the products of human plasma fractionation. xii. the use of concentrated normal human serum gamma globulin (human immune serum globulin) in the prevention and attenuation of measles. J Clin Invest. 1944;23(4):541-9.
12. Stokes J, Maris EP, Gellis SS. Chemical, clinical, and immunological studies on the products of human plasma fractionation. xi. the use of concentrated normal human serum gamma globulin (human immune serum globulin) in the prophylaxis and treatment of measles. J Clin Invest. 1944;23(4):531-40.
13. Young MK, Nimmo GR, Cripps AW, Jones MA. Post-exposure passive immunisation for preventing measles. The Cochrane Library. 2014.
14. Lessler J, Reich NG, Brookmeyer R, Perl TM, Nelson KE, Cummings DAT. Incubation periods of acute respiratory viral infections: a systematic review. Lancet Infect Dis. 2009;9:291-300.
15. McQuillan GM, Kruszon-Moran D, Hyde TB, Forghani B, Bellini W, Dayan GH. Seroprevalence of measles antibody in the US population, 1999-2004. J Infect Dis. 2007;196(10):1459-64.
16. Centers for Disease Control and Prevention. Documentation and verification of measles, rubella and congenital rubella syndrome elimination in the Region of the Americas: United States national report. 2012 March 28, 2012. http://www.cdc.gov/measles/downloads/Report-elimination-measles-rubella-crs.pdf
